# In vitro–in vivo assessments of apocynin-hybrid nanoparticle-based gel as an effective nanophytomedicine for treatment of rheumatoid arthritis

**DOI:** 10.1007/s13346-023-01360-5

**Published:** 2023-06-07

**Authors:** Reham Mokhtar Aman, Randa Ahmed Zaghloul, Wael M. Elsaed, Irhan Ibrahim Abu Hashim

**Affiliations:** 1 Department of Pharmaceutics, Faculty of Pharmacy, Mansoura University, El-Gomhoria Street, Mansoura, 35516 Dakahlia Egypt; 2 Department of Biochemistry, Faculty of Pharmacy, Mansoura University, El-Gomhoria Street, Mansoura, 35516 Dakahlia Egypt; 3 Department of Anatomy and Embryology, Faculty of Medicine, Mansoura University, El-Gomhoria Street, Mansoura, 35516 Dakahlia Egypt

**Keywords:** Phytopharmaceutical hybrid nanoparticles, Apocynin, Chitosan, A fully randomized design (3^2^), Therapeutic activity, Rheumatoid arthritis

## Abstract

Apocynin (APO), a well-known bioactive plant-based phenolic phytochemical with renowned anti-inflammatory and antioxidant pharmacological activities, has recently emerged as a specific nicotinamide adenine dinucleotide phosphate-oxidase (NADPH) oxidase inhibitor. As far as we know, no information has been issued yet regarding its topical application as a nanostructured-based delivery system. Herein, APO-loaded Compritol^®^ 888 ATO (lipid)/chitosan (polymer) hybrid nanoparticles (APO-loaded CPT/CS hybrid NPs) were successfully developed, characterized, and optimized, adopting a fully randomized design (3^2^) with two independent active parameters (IAPs), namely, CPT amount (X_A_) and Pluronic^®^ F-68 (PF-68) concentration (X_B_), at three levels. Further in vitro–ex vivo investigation of the optimized formulation was performed before its incorporation into a gel base matrix to prolong its residence time with consequent therapeutic efficacy enhancement. Subsequently, scrupulous ex vivo–in vivo evaluations of APO-hybrid NPs-based gel (containing the optimized formulation) to scout out its momentous activity as a topical nanostructured system for beneficial remedy of rheumatoid arthritis (RA) were performed. Imperatively, the results support an anticipated effectual therapeutic activity of the APO-hybrid NPs-based gel formulation against Complete Freund’s Adjuvant-induced rheumatoid arthritis (CFA-induced RA) in rats. In conclusion, APO-hybrid NPs-based gel could be considered a promising topical nanostructured system to break new ground for phytopharmaceutical medical involvement in inflammatory-dependent ailments.

**Graphical Abstract:**

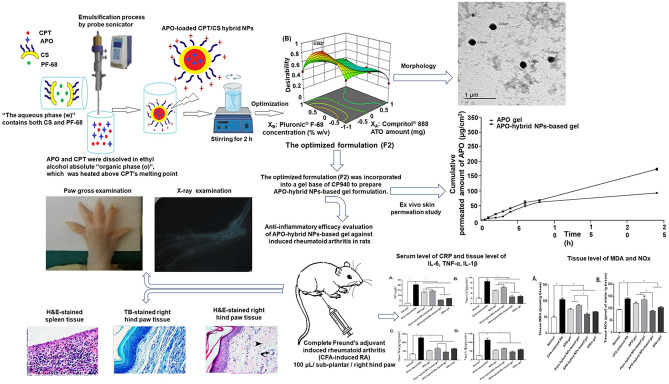

**Supplementary Information:**

The online version contains supplementary material available at 10.1007/s13346-023-01360-5.

## Introduction

Rheumatoid arthritis (RA) is a chronic systemic autoimmune inflammatory disease that warrants innovative treatment development. It is distinguished by inflammation of the synovial membrane, which causes progressive destruction of articular cartilage, joint infiltration, bone erosion and related deformities. Although the scrupulous cause remains unknown, its onset is thought to be due to a combination of environmental and/or genetic factors [1]. Complete Freund’s Adjuvant (CFA), a ready-to-use pale yellow solution of inactivated and dried complete fraction of Mycobacterium butyricum emulsified in mineral oil and used as an immunopotentiator, has been known to promote a series of inflammatory reactions that contribute to the development of RA. For decades, its usage to induce either mono- or poly-arthritic rat model is a well-established experimental model that mimics human RA in both inflammatory and nociceptive complications [2].

Mainly, the conventional treatment approaches for RA involve first-line drug administration such as nonsteroidal anti-inflammatory drugs (NSAIDs) and glucocorticosteroids (GCs), which are mostly used to provide symptomatic relief of RA manifestations. However, their prolonged usage has revealed numerous side effects [1].

Since immemorial time, phytopharmaceuticals, plant-derived natural products, have been applied for therapeutic purposes against various diseases. In addition, the application of modern nanotechnology framework to these compounds has promoted innovations and development of new drugs which have attained immense concerns amongst the researchers [3].

**Fig. 1:**
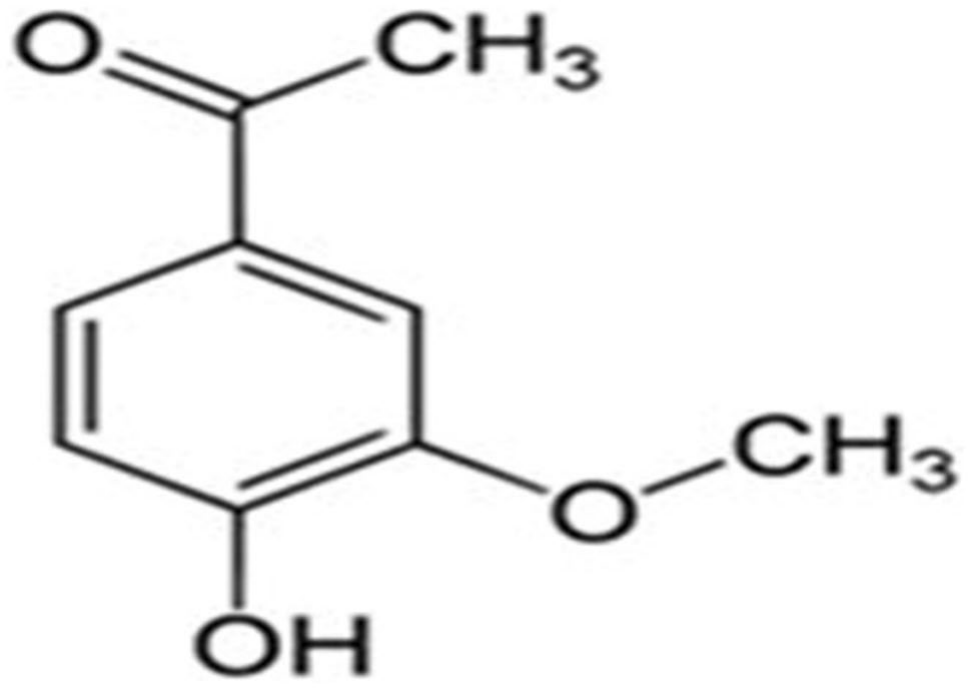
Chemical structure of APO

Apocynin (APO) (Fig. 1) is among bioactive plant-based phenolic phytochemicals which seem expedient for embracing in propitious delivery systems. Originally, APO (3-methoxy-4-hydroxyacetophenone) is a methoxy-substituted catechol isolated either from *Picrorhiza kurroa* (*P. kurroa*) or *Canadian hemp (Apocynum cannabinum)* roots [4]. Reminiscent of other natural phenolic compounds, it boasts specular antioxidant and anti-inflammatory properties related to specialized suppression of nicotinamide adenine dinucleotide phosphate (NADPH) oxidase, besides repression of a set of inflammatory mediators [5, 6]. APO’s efficacious activity has been proved in numerous cell lines and in vivo animal models [7–10]. However, few trials have been reported to develop and evaluate nanometric systems for successful prospective application of APO, with promising augmented therapeutic efficacy [11–18].

Hybrid nanoparticles (NPs) are the newest generation delivery systems that integrate lipid-based and polymeric nanocarriers and combine the benefits rendered by both, which allow incorporation of hydrophilic as well as hydrophobic drugs and improve drug stability besides efficacy [19–22].

Chitosan (CS) is a natural polycationic linear polysaccharide derived from the partial deacetylation of chitin. Based on its promising features, it has consistently inspired research teams to develop novel and more effective parenteral and non-parenteral drug delivery systems. Moreover, low molecular weight (LMW) CS has been reported as non-toxic and non-hemolytic. Additionally, it demonstrates a better biodegradability, biocompatibility and solubility compared to high MW (HMW) CS [23–25].

To the best of our knowledge, no reports have been published yet concerning APO topical application as a nanostructured-based delivery system. Accordingly, such survey outlined the starting-point to direct the current study to prepare a topical nanostructured-based delivery system of APO to reconnoiter its anticipated effectual therapeutic activity against Complete 'Freund's Adjuvant-induced rheumatoid arthritis (CFA-induced RA) in rats.

In this study, optimization of novel APO-loaded Compritol^®^ 888 ATO (lipid)/chitosan (polymer) hybrid nanoparticles (APO-loaded CPT/CS hybrid NPs) was successfully completed by way of following a fully randomized design (3^2^) with two independent active parameters (IAPs) at three levels. The CPT amount (X_A_) and the concentration of pluronic^®^ F-68 (PF-68) (X_B_) were the studied IAPs. The dependent response parameters (DRPs) were hydrodynamic diameter (D_h_), polydispersity index (PI), zeta potential (ζP) and percent encapsulation efficiency (EE %) of APO-loaded CPT/CS hybrid NPs. Before incorporation into a gel base of carbopol 940 (CP940), to improve rheological properties and prolong residence time with subsequent therapeutic efficacy enhancement, the optimized APO-loaded CPT/CS hybrid NPs formulation would be inspected intricately and investigated fundamentally. Consequently, extensive ex vivo–in vivo investigations of APO-hybrid NP-based gel (containing the optimized formulation) was performed to reconnoiter its crucial role as a nanostructured-based topical delivery system for effective treatment of RA were performed.

## Materials and methods

### Materials

CS (CAS Number: 9012–76-4, PCode: 101862602, Lot # STBG9041) with 75–85% degree of deacetylation and LMW (50–190 kDa), APO, besides CFA were purchased from Sigma-Aldrich (Saint Louis, MO, USA). CPT (Batch Number: 175284, Code: 3123) and PF-68 were kindly provided as a gift sample from GATTEFOSSÉ SAS (Saint-Priest Cedex, France) and Amoun Pharmaceutical Industries Company (El Obour city, Cairo, Egypt), respectively. Propyl paraben and methyl paraben were supplied by AppliChem GmbH (Darmstadt, Germany). Carbopol 940 (CP940) was purchased from BDH Chemical Ltd, Liverpool, England. Triethanolamine (TEA) was obtained from Nice Chemicals (Pvt. Ltd., Kerala, India). Chloroform and methanol (HPLC grade, Fischer) were purchased from Cornell lab (Maadi as Sarayat Al Gharbeyah, Maadi, Cairo, Egypt). Analytical grades of ethyl alcohol absolute, glacial acetic acid (99%), sodium chloride (NaCl), propylene glycol, as well as glycerin were procured from El-Nasr Pharmaceutical Chemical Company (Abu Zaabal, Al Qalyubiyah, Egypt).

### Statistical experimental design and data analysis

Over recent years, to overcome the pitfalls of using a conventional screening method, appraising the repercussion of one experimental factor at a time, for NPs formulation optimization, which is tedious and expensive, a statistical experimental design (SED) approach has been widely used. It offers a practical and authenticated analysis of the correlation between the experimental inputs and the measured outputs through the mathematical models’ construction. A fully randomized design (3^2^), as a SED approach, was constructed to study the individual and combined effects of IAPs, specifically; CPT amount (X_A_) as well as PF-68 concentration (X_B_) on different DRPs, namely, D_h_, PI, ζP, and EE % of APO-loaded CPT/CS hybrid NPs. The coded factor levels, embodied in Table 1, were used to probe and exemplify the three levels for each of the two IAPs. By the default design layout, the low levels of the factors were coded as − 1, the medium levels as 0 and the high levels as + 1. Underpinned on the preliminary studies, the actual factor levels, depicted in Table 1, were picked and the optimization approach was ingrained within these domains. A total number of 9 experimental formulae, each with three replicates, were randomized to prepare and optimize APO-loaded CPT/CS hybrid NPs, via employing Design-Expert version 11 (Stat-Ease, Inc., Minneapolis, USA) (Table 2).

**Table 1:** IAPs and their levels used in a fully randomized design (3^2^)

IAPs	Designation	Levels		
		Low ( −)	Medium (0)	High ( +)
A	CPT amount (mg)	75	100	125
B	PF-68 concentration (% w/v)	0.5	0.75	1

**Table 2:** Formulations and properties of APO-loaded CPT/CS hybrid NPs prepared according to a fully randomized design (3^2^)

Formula No	*Coded levels of		D_h_ (nm)	PDI	ζP (mV)	EE %
	A	B				
F1	−	−	650.87 ± 23.76	0.292 ± 0.02	+ 28.50 ± 0.26	48.84 ± 1.65
F2	−	0	418.23 ± 13.48	0.204 ± 0.01	+ 29.00 ± 0.61	34.43 ± 0.21
F3	−	+	651.73 ± 18.85	0.493 ± 0.02	+ 27.67 ± 0.68	38.54 ± 0.18
F4	0	−	539.07 ± 7.19	0.353 ± 0.02	+ 24.97 ± 1.91	45.01 ± 0.04
F5	0	0	738.27 ± 4.45	0.289 ± 0.02	+ 22.53 ± 0.61	38.22 ± 0.07
F6	0	+	732.63 ± 42.96	0.257 ± 0.01	+ 27.30 ± 0.95	50.49 ± 2.32
F7	+	−	866.87 ± 39.35	0.351 ± 0.01	+ 27.50 ± 0.75	54.79 ± 0.11
F8	+	0	787.77 ± 29.92	0.377 ± 0.01	+ 21.77 ± 0.63	38.06 ± 0.28
F9	+	+	610.43 ± 29.15	0.411 ± 0.04	+ 22.43 ± 1.80	46.31 ± 0.05

Each value represents the mean ± SD (*n* = 3)

^*^A, B are CPT amount and PF-68 concentration

Numerous empirically obtained statistical parameters, namely, adjusted coefficients of determination (adjusted *R*^2^), predicted coefficients of determination (predicted *R*^2^), the Fisher model value (*F* value), and the probability value (*p* value) were compared to select the best mathematical regression model fitting the studied DRPs [26, 27]. The adequate mathematical regression equation, predicated on the aforesaid statistical parameters, was selected as follows (Eq. (1)):

1 $$\begin{aligned}Y =\, &\alpha_0 + \alpha_1X_A + \alpha_2X_B + \alpha_3X_{AB} + \alpha_4X_{A}^{2} + \alpha_5X_{B}^{2} \\&+ \alpha_6X_{A}^{2}X_B + \alpha_7X_{A}X_{B}^{2} + \alpha_8X_{A}^{2}X_{B}^{2}\end{aligned}$$

where *Y* is the DRP; α_0_ is the arithmetical average response of the 9 experimental formulae; α_1_ to α_8_ are the linear, interaction, as well as nonlinear coefficients; and *X*_A_ and *X*_B_ are the IAPs.

### Preparation of APO-loaded CPT/CS hybrid NPs

In preparing APO-loaded CPT/CS hybrid NPs, single emulsion-solvent evaporation technique (o/w) was pursued as previously described [21] with slight modifications. Concisely, a total drug quantity (APO_total_ equals 30 mg) was weighed and dissolved in 2 mL ethyl alcohol absolute using an ultrasonic bath (Sonix IV, SS101H 230, ETL Testing Laboratories Inc., USA) for 10 min. After that, an accurately weighed quantity of CPT (75, 100, or 125 mg) was added, and the organic phase (o) was heated above CPT’s melting point (at 77 °C). Subsequently, such organic phase (o) was emulsified with the aqueous phase (w), 10 mL aqueous acetic acid (1% (v/v)) containing both CS (0.1% w/v) and PF-68 (0.5, 0.75, or 1% w/v), and sonicated immediately employing an ultrasonic probe (Model CV 334, Serial Number: 2013020605) attached to a homogenizer (Sonics Vibra-cell™, Model VC 505, Sonic & Materials, INC., USA) in an ice bath under the following conditions: (amplitude: 90%, pulser: 1 s ON, 1 s OFF, timer: 6 min). The volatile organic solvent was completely evaporated via magnetic stirring (Magnetic stirrers, HPS-20D, Taisite Lab Science Inc., USA) at room temperature for 2 h.

Then, the clear supernatant containing free drug (APO_free_) was detached from the prepared APO-loaded CPT/CS hybrid NPs using centrifugal concentration at 10,000 rpm and 4 °C for 1 h (ACCULAB Cooling centrifuge, CE16-4X100RD, USA) using Amicon^®^, 4 mL and 10 kDa cutoff units, Ultra-4 Centrifugal Filter Units (Bioscience Research Reagents, Merck Co., California, USA). Furthermore, the concentrated APO-loaded CPT/CS hybrid NPs were washed with deionized water (DW), resuspended in DW, lyophilized (SIM FD8-8 T, SIM international, USA), and then maintained at 4 °C for further elaborations. The collected supernatant would be kept-up for determination of the EE % of the drug. Blank hybrid NPs analogous to each and every one of the formulae, without APO in the organic phase (o), were prepared similarly.

### Physicochemical evaluation of APO-loaded CPT/CS hybrid NPs

All the prepared formulae were subjugated to physicochemical characterization for different DRPs, including D_h_, PI, ζP, and EE %.

### D_h_ and PI analysis

Adopting the dynamic light scattering (DLS) mechanism, all the recently made APO-loaded CPT/CS hybrid NPs formulae, following pertinent admixing with DW (1:80 v/v) at 25 °C and sonication for 10 min, were evaluated in triplicates for the average D_h_ as well as PI, utilizing Zetasizer Nano ZS (Malvern Instruments, Malvern, UK).

### ζP

ζP is the key parameter that helps assessing the stability of colloidal dispersions. Espousing the electrophoretic light scattering (ELS) technique, the aforesaid diluted samples for all the freshly prepared formulae, following the same dilution and temperature conditions for measuring D_h_ and PI, were evaluated in triplicates for ζP using Malvern Zetasizer Nano ZS.

### EE %

An indirect method was complied to measure EE % for all the formulae. The assembled supernatant containing APO_free_, after centrifugal concentration at 10,000 rpm and 4 °C for 1 h, was appropriately diluted with DW. The free drug was quantified by ultraviolet/visible (UV–Vis) spectroscopy, against the supernatant of blank hybrid NPs corresponding to each formula, at λ_max_ 276 nm (JENWAY 6850, UV–Vis double beam spectrophotometer, UK). The EE % was calculated for each formula using Eq. (2) [21]:

2 $$\mathrm{EE} \% = [(\mathrm{APO}_{\mathrm{total}}-\mathrm{APO}_{\mathrm{free}})]/\mathrm{APO}_{\mathrm{total}}] \times 100$$

### Numerical optimization based on the DFA

Numerical optimization, employing the desirability function approach (DFA), was utilized to pick up the ideal proportions of the operating IAPs to attain the demanded DRPs. Optimization was executed to capture X_A_ and X_B_ levels that minimize both D_h_ and PI, and maximize ζP while keeping EE % within the range of the obtained responses. In addition, the optimized formulation (F2) was picked on a predetermined basis, besides good desirability.

### Characterization of formula 2 (F2) of APO-loaded CPT/CS hybrid NPs

#### Fourier‑transform infrared spectroscopy (FT‑IR) analysis

FT-IR is a versatile, non-perturbing, powerful technique that could be used to obtain information about the structural properties of molecules. FT-IR analysis for APO, CPT, PF-68, CS, their physical mixture (PM) conformed to the optimized formulation as well lyophilized blank and medicated CPT/CS hybrid NPs (F2) was performed using FT-IR spectrophotometer (Nicolet™ IS10™, Thermo Fisher Scientific Instruments Corporation, Madison, Wisconsin, USA). Each sample was homogeneously grinded with potassium bromide (KBr) (10:100), pressurized into discs and scanned (thirty-two scans) over a wavenumber range of 4000 to 500 cm^−1^. Finally, the respective peaks were recorded, displayed, and analyzed using FT-IR data processing software (OMNIC version 8).

#### Differential scanning calorimetry (DSC) studies

As a complementary technique, DSC is a very simple and useful one which provides valuable information concerning the thermodynamic analysis of loaded hybrid NPs [21]. Thermal behaviors of APO, CPT, PF-68, CS, and their PM conformed to the optimized formulation along with lyophilized blank as well as medicated CPT/CS hybrid NPs (F2) were evaluated utilizing a DSC thermal analyzer (LABSYS evo TG–DTA/DSC, Setaram Corp., Caluire, France) calibrated with indium as the reference standard. Accurately weighed samples, constantly purged with inert nitrogen gas at a flow rate of 20 mL/min in hermetically closed aluminum pans, were separately heated at a temperature range and heating rate of 30–400 °C and 10 °C/min, respectively. Ultimately, the display and analysis of data curves automatically via Calisto data treatment software was accomplished.

#### Powder x-ray diffraction (P-XRD) studies

P-XRD is one such analytical technique that offers the advantage of simultaneously characterizing the crystallinity and/or amorphization of the examined precursors besides end products. Utilizing Diano X-ray diffractometer (USA) equipped with Co-Kα radiation, the P-XRD diffraction patterns of APO, CPT, PF-68, CS, their PM conformed to the optimized formulation along with lyophilized blank as well as medicated CPT/CS hybrid NPs (F2) were determined. The assessment was carried out by employing the following circumstances: scanning range (3–50° at 2-Theta (2θ) angle), current (9 mA), and voltage (45 kV).

#### Morphology

Utilizing transmission electron microscopy (TEM) (JEOL JEM-2100, JEOL Ltd., Tokyo, Japan), the inspection of a freshly-made preparation of APO-loaded CPT/CS hybrid NPs (F2) was performed to scrutinize the surface morphology and approximate the size.

A drop of the NPs colloidal dispersion, appropriately admixed with DW, was ultrasonically homogenized for 3 min and cast out onto a TEM grid (carbon-coated copper one). At room temperature, after using a filter paper to wipe out the remaining dispersion, such TEM grid was subjected to air-drying. Then, direct imaging of unstained nanostructures was accomplished via TEM at 160 kV. Ultimately, image capture and analysis process were accomplished using imaging viewer software (Gatan Microscopy Suite Software, version 2.11.1404.0).

### Ex vivo skin permeation study

#### Preparation of rat skin

Directly before performing the experiment, hair from the abdomen, of each newborn Wistar albino rat (2 weeks old), was removed using clippers. After 24-h period, the skin integrity was attentively scrutinized for any damaged skin, the rats were abandoned by spinal dislocation, the full-thickness abdominal skins were excised, and the subcutaneous fat was removed. Eventually, the skin was quite washed with DW, cut into appropriate size matching with the utilized diffusion cell and drenched all-night in 0.9% NaCl solution, as an isotonic saline, at refrigerator degrees. Prior to the experiments, the skin was allowed to match the room temperature [28–30].

#### Skin permeation experiment

Locally fabricated horizontal Franz diffusion cells, having a surface area of 4.91 cm^2^, were utilized to perform such an experiment for APO suspended in propylene glycol (control) as well as the optimized formulation (F2). Each sample (equivalent to 3.10 ± 0.115 mg APO) was introduced to the stratum corneum (SC), comprising the donor chamber of the excised rat skin, while the dermal side faced the receptor one that was filled with 50 mL phosphate buffer (PB (pH 7.4)) and shaken at 100 rpm in a shaking incubator (GFL Gesellschaft für Labortechnik, Burgwedel, Germany) maintained at 37 ± 0.5 °C. Such assessment was carried out in triplicate.

At preplanned time intervals (0.5, 1, 2, 3, 4, 6, 8, and 24 h) and to keep up a steady volume during the experiment, aliquots of 3 mL were collected from the receptor compartment and resubstituted with the same volume of PB (pH 7.4). The huddled aliquots were filtered by 0.45 µm membrane filters (EMD Milli-pore, Billerica, MA, USA) and quantified spectrophotometrically for permeated drug amounts using UV–Vis spectrophotometer at 278 nm. To eliminate whichever interference deriving either from rat skin or formula constituents, the same protocol was fulfilled utilizing plain CPT/CS hybrid NPs, as blank, corresponding to the investigated medicated formula (F2).

#### Skin permeation parameters

The cumulative permeated APO amount, in the receptor compartment, through the rat skin per unit area (Q) (μg/cm^2^) was plotted against time (t) (h) for the screened samples. The skin permeation parameters, specifically the cumulative permeated amount of APO per unit area after 24 h, steady-state flux, permeability coefficient, and enhancement ratio of flux, being expressed as (Q_24h_) (μg/cm^2^), (J_ss_) (μg/cm^2^.h), (K_p_) (cm/h), and (ER_flux_), respectively, were estimated as reported [29–31].

#### Preparation of APO gel and APO-hybrid NP-based gel

To attain improved rheological properties, prolonged residence time with succeeding enhanced therapeutic efficacy at the application site besides ameliorated patient acceptability and applicability, the optimized formulation (F2) was integrated in a gel base of CP940 (0.5% w/w) to prepare APO-hybrid NPs-based gel formulation. Besides, APO gel formulation (containing the free drug) was also prepared.

Succinctly, for preparing APO gel formulation, the specified weight of CP940 (0.5% w/w) was sprinkled on a little indefinite quantity of DW and left overnight to hydrate. Thereafter, APO's specified concentration (0.2% w/w) was triturated with propylene glycol (10% w/w), added to the gel, succeeded by incorporation of glycerol (10% w/w) and remnant components (methyl and propyl parabens; 0.05% w/w for each) under constant magnetic stirring. DW was further added for adjustment of the final weight of the gel to 100 g. Ultimately, the final gel was neutralized via dropwise addition of TEA, neutralizing agent, to achieve a transparent semisolid gel of pH value of around 6.5 using a digital pH meter (JENWAY 3540 Bench Combined Conductivity/pH Meter, Cole-Parmer, Stone, Staffordshire, ST15 OSA, UK). With respect to preparation of APO-hybrid NPs-based gel formulation, an identical approach was embraced for integrating the optimized formulation (F2) commensurate with the appointed concentration of drug in the gel matrix [30, 32].

### Evaluation parameters of APO gel and APO-hybrid NP-based gel

#### Appearance and color

The homogeneity, appearance, color, and presence of any aggregates or lumps were visually scrutinized for the prepared gel formulae.

#### Viscosity measurement

Cone and plate rotary viscometer (Haake Inc., Vreden, Germany) was utilized to measure the viscosity of the prepared gels. The upper cone was adjusted, while the attested formulations were spread attentively onto the lower stationary plate of viscometer with a diameter of 2.9 cm. Then, samples were allowed to equilibrate for 5 min to attain the running ambient temperature, the speed value “n” was maintained at 256 rpm and the torque value was acquainted from the scale grade “S.” For viscosity calculation, the following Eq. (3) was employed:

3 $$\eta = \frac{G.S}{n}$$

where *η* is the viscosity in millipascal second (1 mPa s = 1 centipoise (cP)), *G* is the instrumental factor = 14,200 (mPa s/scale grade min), *S* is the torque (scale grade), and *n* is the speed (rpm). Measurements were performed in triplicate for each sample.

#### pH measurement

The pH of the prepared gel formulae was measured using a digital pH meter (JENWAY 3540 Bench Combined Conductivity/pH Meter, Cole-Parmer, Stone, Staffordshire, ST15 OSA, UK).

#### Drug content

In stoppered volumetric flasks, 1 g of each one of the two medicated gel formulae besides their corresponding plain ones was dissolved in 25 mL of organic solvent (a mixture of chloroform and methanol in a ratio of 1:1 v/v) and sonicated for 20 min to draw out the drug. Then, the resulting solutions were filtered, using Whatman^®^ filter paper followed by 0.45 µm membrane filter, and suitably diluted with the organic solvent mixture. Finally, APO concentration, for each medicated gel formulation against its corresponding plain, was estimated via UV–Vis spectrophotometer at 270 nm.

### Ex vivo skin permeation study for APO gel and APO-hybrid NPs-based gel

The same procedures of the ex vivo permeation study, even as mentioned earlier in the “Ex vivo skin permeation study ” section, for formula 2 (F2) of APO-loaded CPT/CS hybrid NPs characterization, were embraced for APO gel and APO-hybrid NPs-based gel (≃ 1.5 gm gel), in triplicate. Similarly, the skin permeation parameters were estimated as aforementioned detailed.

### Kinetic analysis of permeation data

Ex vivo permeation data of the optimized formulation (F2), APO gel, and APO-hybrid NPs-based gel were analyzed using different kinetic models including zero order, first order, and Higuchi’s square root models [33]. Moreover, the proper drug release mechanism was assessed via application of the Korsmeyer-Peppas model, first 60% permeation data, according to the following Eq. (4) [34, 35]:

4 $$M_t/M_\infty = kt^n$$

where *M*_*t*_/*M*_∞_, *k*, *t*, and *n* denote the fraction of drug released, the kinetic constant, the release time, and the characteristic diffusional exponent for the release mechanism, respectively.

### Storage stability study

Freshly prepared APO-hybrid NPs-based gel formulations were packed in glass bottles and subjected to stability study under different storage conditions, namely, refrigerated (4 ± 1 °C) and ambient (25 ± 2 °C/60 ± 5% relative humidity; RH) conditions over a period of 6 months. Physical evaluation of the samples was achieved by visual scrutinization of any change in appearance, color, and/or presence of any aggregates or lumps. Moreover, the stability of APO-hybrid NPs-based gel was assessed in terms of drug retention %, viscosity measurement and pH measurement at zero time (freshly prepared formulation at production day), and after storage periods of 1, 3, and 6 months as described above [36].

### Anti-inflammatory efficacy of APO-hybrid NPs-based gel against CFA-induced RA in rats

#### Experimental design

An in vivo evaluation of APO-hybrid NPs-based gel was investigated for its anti-inflammatory effect in CFA-induced RA in rats. The study protocol was reviewed and accepted by the ethical committee of Faculty of Pharmacy, Mansoura University, Mansoura, Egypt, following the “Principles of Laboratory Animal Care, National Materials Institute of Health Publication (No. 85–23, revised 1985)” (Ethical Approval Code 2022–131), and according to the Animal Research: Reporting of In Vivo Experiments (ARRIVE) guidelines.

### Experimental outline

The study was conducted on thirty adult male Wistar rats (160–240 g). Rats were maintained under standard conditions of animal care, temperature (23 ± 2 °C), and steady light/dark cycles. They were randomized into metallic cages (*n* = 5). Rats were permitted free access to water and standard animal food throughout the experiment. Following a week from their housing, a vernier caliper (Tricle Brand, Shanghai, China) was utilized to capture the right paw measurements of these rats. They were anesthetized with ketamine chloride [40 mg/kg/intraperitoneal (i.p.)] and divided into two major groups:

1. Normal control group (*n* = 5); rats received a sub-plantar injection of 100 μl physiological saline in the right hind paw.
2. Arthritic group (*n* = 25); rats were given CFA as 100 μl in the sub-plantar region of the right hind paw (day 0) [37].

Animals were inspected daily for the development of localized edema (swelling). Then, on the 14^th^ day, the 25 rats given CFA, in the arthritic group, were randomly realigned into the following five groups (*n* = 5):

- CFA-induced RA group.
- APO gel group; rats were treated topically with APO gel as 15 mg/kg/twice/day.
- Plain hybrid NPs-based gel group; rats were treated topically with plain hybrid NPs-based gel, corresponding to APO-hybrid NPs-based gel, twice/day.
- APO-hybrid NPs-based gel group; rats were treated topically with APO-hybrid NPs-based gel as 15 mg/kg/twice/day.
- Olfen^®^ gel group; rats were treated topically with a commercial gel containing diclofenac sodium (Olfen^®^ gel, Medical Union Pharmaceuticals Company (MUP), Egypt) as 4.5 mg/kg/twice/day [37].

The groups were treated topically with the calculated dose, twice daily, from day 14 till day 28, by 50-time motion with the tip of the finger. The treatment protocol is summarized in Fig. S1 (Supplementary material).

### Paw thickness, body weight, arthritic score, gross morphology, and X-ray examination

Rat paw thickness, using digital vernier calipers, and body weight, using a digital balance (Kent Scientific Corporation, Torrington, USA), were assessed on days 0, 7^th^, 14^th^, 21^th^, and 28^th^. The changes in paw thickness, as well as body weight for each rat, were calculated as follows (Eqs. (5–6)) [38]:

5 $$\begin{aligned}\%\ {\mathrm{Change\ in\ paw\ thickness}} = \frac{\mathrm{(Final\ right\ paw\ thickness - Initial\ right\  paw\ thickness)}}{\mathrm{Initial\ right\ paw\ thickness}} \times 100\end{aligned}$$

6 $$\begin{aligned}\%\ \mathrm{Change\ in\ the\ body\ weight} = \frac{\mathrm{(Final\ Body\ weight - Initial\ Body\ weight)}}{\mathrm{Initial\ Body\ weight}} \times 100\end{aligned}$$

Besides, the arthritic score was evaluated on the 28^th^ day by giving the severity of arthritis a score (scale 0–4) for the two hind paws, with a maximum score of eight. The scoring was as follows: 0, indicating no swelling or erythema; 1, indicating mild digital swelling or erythema; 2, indicating moderate swelling; 3, indicating severe swelling and erythema of the ankle; and 4, indicating ankylosis or inability to bend the ankle [39]. Finally, rats were anesthetized with ketamine chloride (40 mg/kg/i.p.) and their hind paws were photographed for gross morphology investigation with subsequent X-ray examination (55 kV peak, 50 mA with exposure time of 5 s) [40, 41].

### Collection of the biological samples “blood as well as tissues” and biochemical analysis

Blood samples were collected from the retro-orbital plexus following an X-ray examination. After blood coagulation under ambient conditions and centrifugation at 5000 rpm for 15 min (MSE centrifuge, UK), sera were obtained and directly utilized for quantification of C-reactive protein (CRP) (Tina-quant C-reactive protein Gen.3, Roche Diagnostics, USA). Thereafter, rats were cervically decapitated and the right hind paw (5 cm below and above the joints), as well as the spleen, was excised.

Spleen tissues were washed, blot-dried and then weighed for assessment of the spleen index as in the following Eq. (7) [38]:

7 $$\mathrm{Spleen\, index} = \frac{\mathrm{Spleen\, weight\, (g)}}{\mathrm{Rat\, weight\, (g)}} \times 100$$

One set of the collected paw tissues, from all the experimental groups, was preserved in 10% buffered formalin and then fixed in paraffin wax for histopathological examination. The remaining set was homogenized in phosphate buffer saline (PBS), pH 7.4, 10% w/v, and centrifuged to collect the supernatant. The homogenates were frozen at − 80 °C for further analysis.

Samples of right paw homogenate were utilized for the estimation of the levels of malondialdehyde (MDA), and nitric oxide (NOx) by commercially available kits (MD 25 29 and NO 25 33, Biodiagnostic Co., Giza, Egypt) according to the manufacturers’ instructions. Likewise, another homogenate samples were utilized for the assessment of rat tumor necrosis factor-α (TNF-α), interleukine-1β (IL-1β), and interleukin 6 (IL-6) by commercially available enzyme-linked immunosorbent assay (ELISA) kits (Cat. No. abx050220, Abbexa, Ca, UK, and E0119 Ra and E0135Ra, BT LAB Co., Shanghai, China, and Cloud Clone Co., Wuhan, China, respectively) according to the manufacturers’ instructions.

### Histopathological examination

Preserved right paw parts in paraffin wax were cut into 5-μm-thick coded sections and then stained with either Mayer’s hematoxylin and eosin (H&E) or toluidine blue (TB). Histological alterations were observed and photographed using an Olympus^®^ digital camera attached to an Olympus^®^ light microscope (Shinjuku co., Tokyo, Japan) by a skilled pathologist unaware of the coding system of the different groups. The histological changes were evaluated via the determination of the relative area of dermal layers in paw tissue quantitatively by Image J software (1.52a, NIH, USA). Histopathological evaluation of the severity of joint inflammation was scored using four parameters: cartilage/bone erosion, pannus development, inflammation, and synovial hyperplasia. Each histopathological parameter was scored from 0 to 4, yielding a maximal value of 16 for each joint, with higher scores indicating more severe disease.

For paw edema, the histological changes were evaluated with relative areas of the dermis in paw tissue quantitatively by ImageJ software (*n* = 5).

### Statistical analysis

The in vitro besides ex vivo data were displayed as mean ± standard deviation (SD), while in vivo ones were expressed as mean ± standard error of the mean (SEM). One-way analysis of variance (ANOVA) followed by Tukey–Kramer multiple comparisons test was utilized for statistical scrutinization of the in vitro data, while the Student’s *t*-test (unpaired *t*-test) was adopted for ex vivo data analysis. The dissimilarities between the different rat groups were evaluated by one-way ANOVA followed by Tukey’s multiple comparison post hoc test. Instead, the nonparametric data, for histopathology, arthritic scoring, % area of paw edema, and spleen index, were analyzed using the Kruskal–Wallis test followed by the uncorrected Dunn multiple comparison tests, and the data were represented as median and interquartile range. The statistical significance was judged at (*p* < 0.05). To accomplish such statistical inference, GraphPad Prism versions 5.00 and 8.0.2 (GraphPad Software, Inc., La Jolla, CA, USA) were employed. The applied SED approach (3^2^ fully randomized design) was appraised concerning statistical influence utilizing ANOVA by Design-Expert version 11 (Stat-Ease, Inc., Minneapolis, USA). Statistically significant *F *values (*p* < 0.05) besides adjusted *R*^2^ ranging from 0.8 to 1.0 were the criteria for evaluating the selected mathematical regression model, as reported earlier [13, 42]. Moreover, the effect of IAPs on the DRPs was presented as contour plots as well as response surface plots created by changing X_A_ as well as X_B_ along the studied domain.

## Results and discussion

### Preparation, characterization, and optimization of APO-loaded CPT/CS hybrid NPs

In the framework of this contemporary study, APO-loaded CPT/CS hybrid NPs were successfully prepared by a single emulsion-solvent evaporation technique (o/w). The superior attributes of the combination of both lipid and polymeric carriers offered by the hybrid NPs, could promote the current nanotherapeutic systems advancement in numerous applications, including drug delivery, targeting, and diagnostic ones [20]. Hence, as far as we are aware, this is the foremost endeavor for APO enwrapping in hybrid NPs to capitalize on their merits.

Furthermore, for amending the drug absorption and accordingly its therapeutic efficacy, critical DRPs considerations including minimum D_h_ and PI, maximum ζP and reasonable EE % within the range of the obtained responses need to be considered. A fully crossed design (3^2^) afforded appropriate management of these considerations, during the initial phase of development, via precise analysis of IAPs and their interactions. Eventually, optimization was executed to obtain the levels of IAPs, namely, X_A_ and X_B_, which minimized both D_h_ and PI and maximized ζP while keeping EE % within the range of the obtained responses.

### D_h_ and the PI

Not only the knowledge of NPs’ optimal D_h_ is a paramount requirement, but also the breadth of their size distribution. Generally, D_h_ ranging from 10 to 600 nm has been reported to allow drug delivery of the encapsulated materials through the skin layers [43]. PI, used to delineate the degree of nonuniformity of the size distribution of particles, is a unitless scaled number, ranging from 0.0 to 1.0 and calculated from a two-parameter fit to the correlation data (the cumulants analysis). Controlling and validating these indices are substantial for the efficacious therapeutic applications of nanocarriers. Moreover, both of which have to be thoroughly studied to determine the colloidal dosage form stability upon storage.

The average D_h_ and PI values of all the prepared NPs formulae (F1–F9) are presented in Table 2. Such aforementioned values were of 418.23 ± 13.48 to 866.87 ± 39.35 and 0.204 ± 0.01 to 0.493 ± 0.02, respectively. Lower PI values (< 0.5) depict better distribution and are referred to as a homogenous hybrid NPs dispersion (monodisperse). Furthermore, they are imperative for evaluating the stability of a colloidal dosage form upon storage [43].

The optimized mathematical regression models for these DRPs are symbolized as follows (Eqs. (8–9)):

8 $$\begin{aligned}D_h =\,& +738.27 +184.77X_A +96.78X_B -64.33X_{AB}\\& -135.27X_{A}^{2} -102.42X_{B}^{2} -160.67X_{A}^{2}X_B\\& -141.09X_AX_{B}^{2} +194.39X_{A}^{2}X_{B}^{2}\end{aligned}$$

where *F* = 85.35, *p* < 0.0001, and adjusted *R*^2^ = 0.9657

9 $$\begin{aligned}\mathrm{PDI} =\, &+0.2887 +0.0865X_A -0.0482X_B -0.0353X_{AB}\\& +0.0015X_{A}^{2} +0.0162X_{B}^{2} +0.1134X_{A}^{2}X_B\\& -0.0922X_AX_{B}^{2} +0.0807X_{A}^{2}X_{B}^{2}\end{aligned}$$

where *F* = 43.35, *p* < 0.0001, and adjusted *R*^2^ = 0.9339

Mindful inspection of the two demonstrated equations discloses that the CPT amount effect, linear one (X_A_), owns the highest coefficient “positive effect” on DRPs such as D_h_ and PI. Discordantly, PF-68 concentration (X_B_) has a “positive effect” on D_h_, while it has a “negative one” on PI. Interaction between both CPT and PF-68 (X_AB_) is antagonistic towards D_h_ and PI, while a non-linear one (X_A_^2^X_B_^2^) is synergistic.

Supposedly reported by [44, 45] increasing CPT amount (X_A_), known to have high viscosity and high melting point, prompts an increase in the D_h_ as a consequence of the difficult disruption of a hot oil droplet, retarded breakdown rate as well as delayed lipid crystallization. Likewise, a wide range of size distribution, and higher PI values, were asserted to occur when the lipid content was increased [46, 47]. Presumably, a high level of PF-68 (X_B_), being the surface-active agent, might lead to an increase in the viscosity of the aqueous phase (w), a decrease in the droplets breakdown rate into smaller ones, and subsequent D_h_ enlargement. Also, flocculation, owing to the dehydration of PF-68 chains, and reduced steric stabilization efficiency may account for such enlargement [48]. Instead, an improvement in homogeneity was accompanied.

Table 2 bares that an increase in CPT (X_A_) from 75 to 100 and 125 mg while maintaining the X_B_ constant (F2, 5 and 8) increased D_h_ and PI, while keeping X_A_ constant and increasing PF-68 (X_B_) from 0.5 to 0.75 and 1% w/v (F4, 5 and 6) increased D_h_ values and decreased PI ones.

**Fig. 2:**
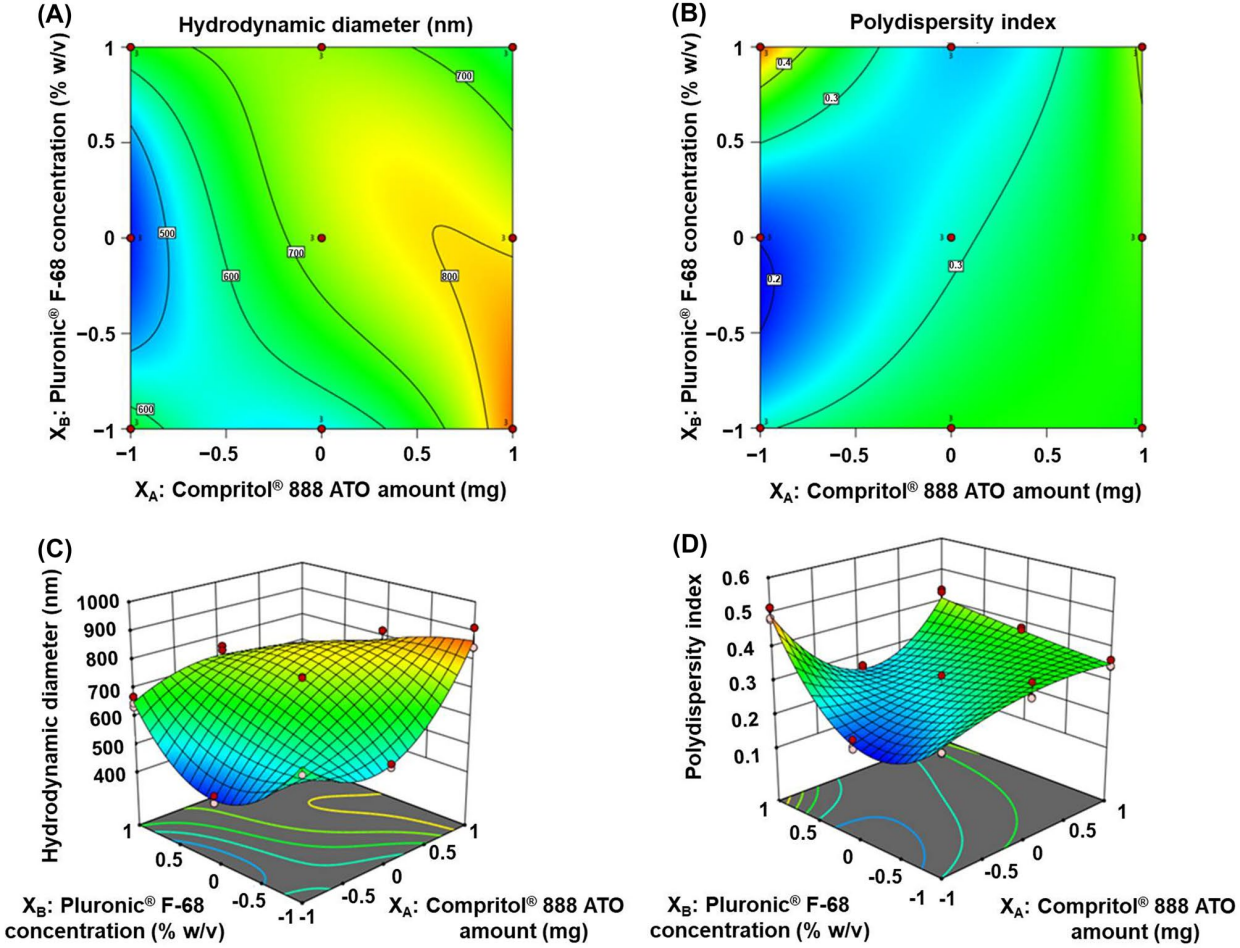
Contour (**A** and **B**) as well as response surface (**C** and **D**) plots representing the effect of the interaction between CPT amount (X_A_) and PF-68 concentration (X_B_) on D_h_ and PI, respectively

Nonetheless, to consider easier elucidation of IAPs impact on DRPs, Design Expert software utilizes the coded-based equation to plot various graphs for D_h_ and PI. The contour (Fig. 2A and B) as well as response surface (Fig. 2C and D) plots, respectively, depicts the diversities in the aforesaid DRPs against the two IAPs, namely X_A_ (CPT) and X_B_ (PF-68). Considering the contour plots, it is obvious that the lowest D_h_ and PI values are achieved at a low level of CPT (X_A_) and a medium level of PF-68 (X_B_). Thus, it can be concluded that, in such study, CPT (X_A_) and PF-68 (X_B_), at their low and medium levels, respectively, are requisite to prepare hybrid NPs with the lowest D_h_ besides PI (F2).

### ζP

NPs’ surface is inevitably charged. A potential difference arises between the NPs’ surface and the surrounded bulk fluid, called ζP, which has a very intimate relation to their mucoadhesive property, cellular uptake ability, and long-term stability. Therefore, it is recognized that positively charged NPs favor adhesion to the negatively charged mucoproteins found throughout the different body mucosae, including the skin, thus facilitating their transport into the skin via the intra- or intercellular pathways. It is worthwhile to remember that for provision of very good stability in the dispersion medium, absolute ζP value below − 30 mV or above + 30 mV is required [49, 50]. The optimized mathematical regression model for ζP is signified hereby (Eq. (10)):

10 $$\begin{aligned}\zeta P =\, &+22.53 -3.62X_{A} +1.17X_{B} -1.06X_{AB} +2.85X_{A}^{2} \\&+3.60X_{B}^{2} -2.64X_{A}^{2}X_B +2.06X_AX_{B}^{2} -2.46X_{A}^{2}X_{B}^{2}\end{aligned}$$

where *F* = 20.33, *p* < 0.0001, and adjusted *R*^2^ = 0.8657

Here, the average ζP values of all the prepared NPs formulae are displayed in Table 2. Aforesaid positively charged values ranged from + 21.77 ± 0.63 to + 29.00 ± 0.61 (F2 and 8, Table 2). Such a proclivity might be ascribed to the supremacy of freely ionized amino groups of CS, in the aqueous phase (w), over the ionized carboxylic acid groups of CPT in the organic phase (o).

**Fig. 3:**
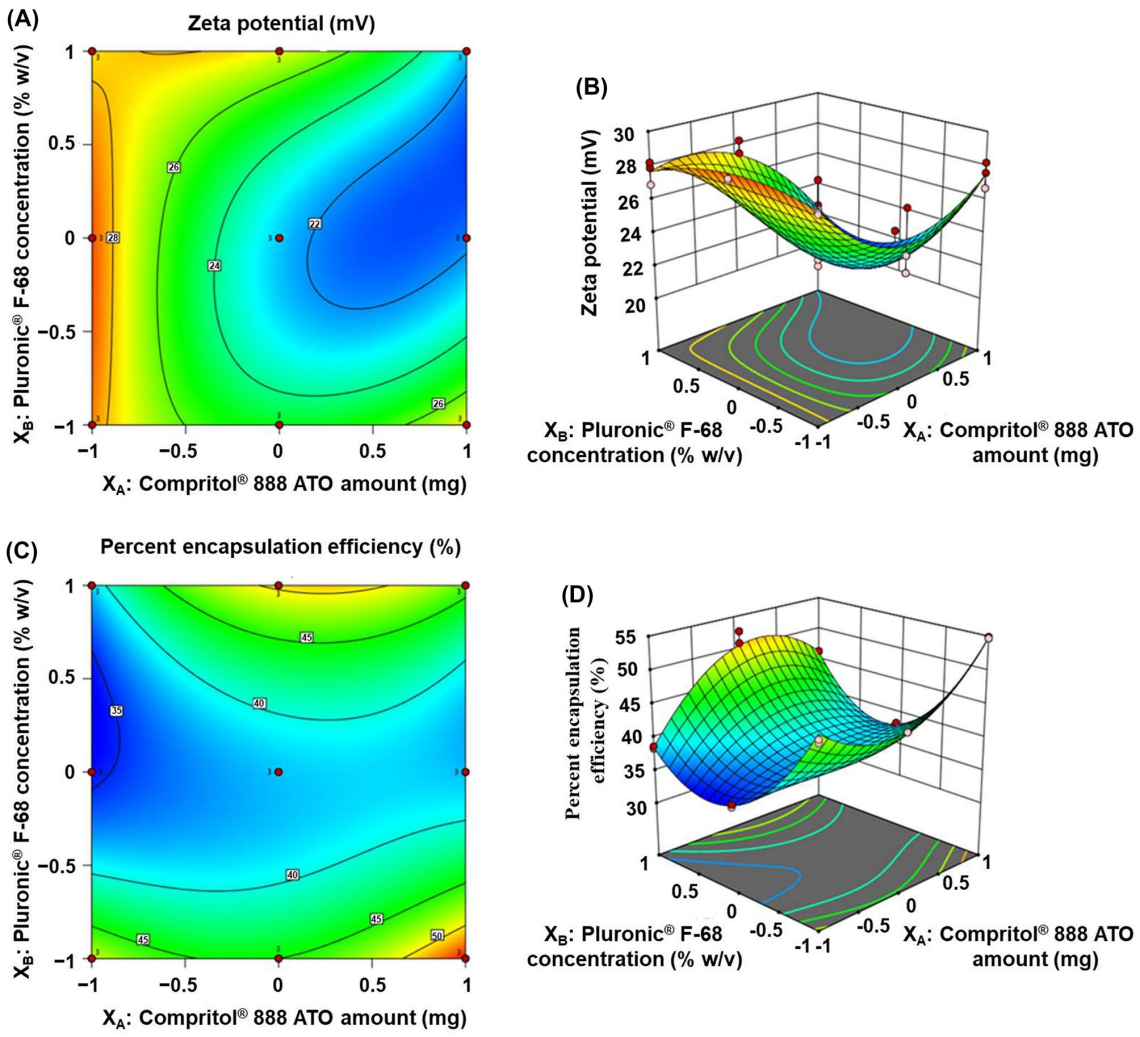
Contour (**A** and **C**) as well as response surface (**B** and **D**) plots representing the effect of the interaction between CPT amount (X_A_) and PF-68 concentration (X_B_) on ζP and EE %, respectively

The above-named equation shows that the linear effect of CPT amount (X_A_) has the highest coefficient “negative effect” towards ζP, while PF-68 concentration (X_B_) has an opposite effect “positive linear one (X_B_).” Moreover, the interaction between both of which (X_AB_) is antagonistic. Increasing CPT amount (X_A_), with subsequent increment of the available negatively ionized carboxylic acid groups on its surface, persuades an ultimate decrease in the NPs’ positive ζP values. Similarly, a simultaneous increase in both CPT (X_A_) and PF-68 (X_B_) would significantly decrease the ζP values of hybrid NPs owing to much more ionization of CPT carboxylic acid groups. Contrarily, an increase in the surface-active agent “PF-68” layer thickness decreases the negative charge density, as a consequence of the outward shift of the slipping plane at which the ζP was measured, with a subsequent predominance of protonated amino groups on the surface of NPs and higher recorded ζP values [51, 52]. Penetration besides accumulation in deeper skin areas are potentially augmented via NPs’ acquiring positive ζP values [53]. Table S1 (Supplementary material) outlines the statistical relevance of all IAPs as well as their interactions with ζP and the remainder DRPs. Moreover, equation outcomes are substantiated by graphs depicted in Fig. 3A and B. Considering these plots, it is evident that, CPT (X_A_) and PF-68 (X_B_), at their low and medium levels practiced in such study, respectively, are required to prepare hybrid NPs with maximum ζP (F2).

### EE %

Indeed, such aforenamed DRP is a mandatory measure for appraisal of the effectiveness and reproducibility of the embraced technique. The EE % of APO-loaded CPT/CS hybrid NPs varied from 34.43 ± 0.21 to 54.79 ± 0.11%. The optimized mathematical regression model, delineating the linear, interaction as well as nonlinear effects of the surveyed IAPs on EE %, is illustrated as follows (Eq. (11)):

11 $$\begin{aligned}EE\, \% =\, &+38.22 +1.82X_A +2.74X_B +0.4525X_{AB}\\& -1.98X_{A}^{2} +9.53X_{B}^{2} -7.43X_{A}^{2}X_B \\&+1.61X_AX_{B}^{2} +1.35X_{A}^{2}X_{B}^{2}\end{aligned}$$

where *F* = 138.78, *p* < 0.0001, and adjusted *R*^2^ = 0.9787.

Mindful inspectorial of the aforementioned equation unveils that the effect of PF-68 concentration, either linear (X_B_) or nonlinear (X_B_^2^), linear effect of CPT (X_A_) besides the non-linear effect of their interaction (X_A_X_B_^2^) exhibit the highest positive coefficients “favorable effect” and display statistical significance, as well (Table S1 (Supplementary material)). Presumably, increasing CPT amount confers more space to accommodate an excess amount of the drug throughout hybrid NPs preparation. Additionally, increasing PF-68 concentration enhances the solubilization of drug molecules inside the lipid lattice and at the outer surface of the NPs, hence resulting in an increase in EE % of APO in the APO-loaded CPT/CS hybrid NPs. These results were correlated with reported study [52].

An increment in CPT (X_A_) from 75 to 125 mg while maintaining X_B_ constant (F1, 7, F2, 8, and F3, 9), besides an increase in PF-68 (X_B_) from 0.75 to 1% w/v and keeping X_A_ constant (F2, 3, F5, 6 and F8, 9) increased EE % values (Table 2). Figure 3C and D show contour and response surface plots, respectively, of changes in EE % opposed to variance with the two IAPs, CPT (X_A_) and PF-68 (X_B_), all-around the studied extent.

### Numerical optimization based on the DFA

Concerning numerical optimization, SED is widely practiced with DFA to identify the best formula out of all the prepared formulae. As reported, DFA transforms an estimated dependent response parameter (DRP) into a scale-free value, called desirability with its value between 0 and 1. Then, the overall desirability (D), another value between 0 and 1, is computed by an automatic combination of the individual desirability values for each DRP selected criterion. The overall D score of the contemporary design (Fig. S2 (Supplementary material)) was found to be 0.9627, for F2 which exhibited minimum D_h_ as well as PI, maximum ζP, and EE % value within the range. Values near one imply accurate outputs of the design, whilst those nearby zero designate inaccurate ones [54]. Consequently, the optimized formulation (F2), with low and medium levels of CPT (X_A_) and PF-68 (X_B_), respectively, was exposed to further imposed investigations.

### Characterization of F2 of APO-loaded CPT/CS hybrid NPs

#### FT‑IR analysis

**Fig. 4:**
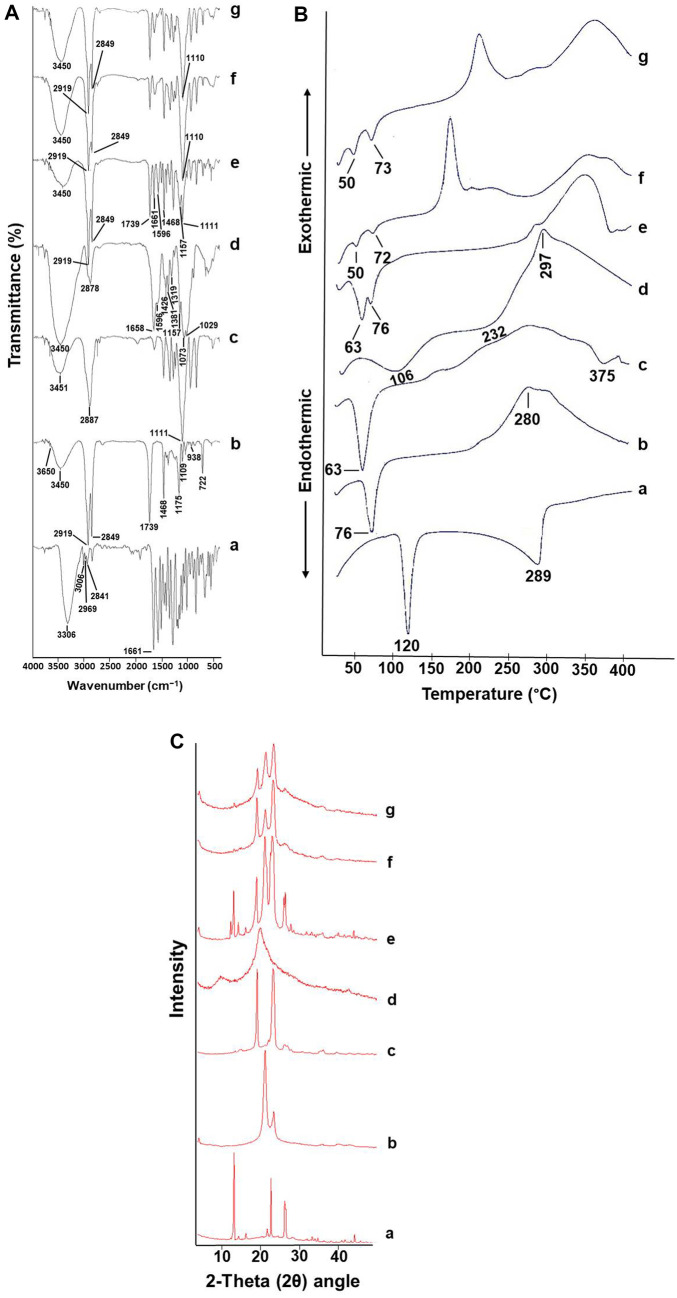
FT-IR spectra (**A**), DSC thermograms (**B**), and XRD scan diffractograms (**C**) of APO (**a**), CPT (**b**), PF-68 (**c**), CS (**d**), their PM (**e**), blank CPT/CS hybrid NPs (**f**), and APO-loaded CPT/CS hybrid NPs (F2) (**g**)

Figure 4A depicts the FT-IR respective peaks of the optimized APO-loaded CPT/CS hybrid NPs formulation (F2) and its components. Regarding the infrared spectrum of APO (a), the distinctive peaks at 3306, 3006, as well as between 2841 and 2969 cm^−1^ were originated from phenolic OH, aromatic‑hydrogen,, and alkane carbon‑hydrogen stretching vibrations, respectively. Besides, stretching vibration of the ketonic carbonyl (–C = O) group was indicated by the distinct band at 1661 cm^−1^ [12, 55]. All of CPT (b) functional groups’ discriminatory bands were disclosed at 3650–3100 (–OH), 2919 and 2849 (alkane –C–H), 1739 (ester carbonyl (–C = O)) as well as between 700 and 1500 cm^−1^ (aliphatic –CH_2_ and –CH_3_) [56, 57]. Regarding PF-68 spectrum (c), the infrared shoulders discerned at 3451, 2887, and 1111 cm^−1^ were allocated to stretching vibrations of (–OH, –CH_3_ and C–O–C), respectively [58, 59]. CS spectrum (d) clarified a characteristic absorption band at 3450 cm^−1^ that was attributed to stretching vibration as well as intermolecular hydrogen bonding of (–OH) besides (N–H) groups. The stretching band of (C–H) from alkyl groups was represented at 2878 cm^−1^, while the bands at 1658, 1596, as well 1319 cm^−1^ exemplified amides I, II, and III, respectively. Otherwise, the bands at 1426 and 1381 cm^−1^ symbolized (–CH_2_) bending besides (–CH_3_) symmetrical stretching, respectively. The anti-symmetric stretching of (C–O–C) bridge was delineated at 1157 cm^−1^. Additionally, the structural vibrations bands, including the (C–O) stretching at 1073 and 1029 cm^−1^, were reminiscent of CS' saccharide skeleton [60, 61].

The characteristic peaks of the individual components, namely, CPT, PF-68, and CS, were observed in the PM spectrum (e), while the particular ones concerning APO were either diminished or even disappeared as a consequence of the dilution effect. Blank (f) and medicated (g) lyophilized CPT/CS hybrid NPs spectra consented with each other, wherever both showed intense peaks at 3450, 2919, 2849, and 1110 cm^−1^ indicating the probability of individual constituents' overlapping. Furthermore, in the spectrum of medicated NPs, in the region of 400–1700 cm^−1^, APO’s fingermark intense peaks faded with consequential verification of its encapsulation in the hybrid matrix. Analogous findings were previously reported [21].

### DSC studies

As stated previously, DSC can be employed to investigate thermodynamic disparities related to morphological changes during the preparation of lipid-based NPs [62].

As illustrated in Fig. 4B, the characteristic two endothermic peaks at 120 and 289 °C, corresponding to the melting point and thermal decomposition of pure APO (a), respectively, appeared indicating its crystalline nature [14]. CPT thermography (b) exhibited a discriminative melting endothermic peak at 76 °C besides an exothermic one at around 280 °C, that could be attributed to lipid recrystallization [63]. Regarding PF-68 thermography (c), a characteristic melting endothermic peak at 63 °C besides a broad one, ascribed to thermal degradation, at 375 °C were observed [64]. Meanwhile, the DSC thermography of CS (d) displayed no sharp peaks, instead, two broad endothermic ones, as well as an exothermic one, were observed. The endothermic peaks were at around 106 and 232 °C ascribing to the idiosyncratic dehydration and melting temperatures of CS, whilst the exothermic one was at 297 °C corresponding to its structural glucosamine units’ decomposition [65].

Concerning the thermography of their PM (e), the discernible melting events of PF-68 and CPT were separately recorded at 63 and 76 °C, respectively [66]. A remarkable absence of those concerning APO (30 mg) and CS (10 mg) might be imputed to peaks’ masking by the comparatively large amounts of both PF-68 and CPT (75 mg) [67].

Intriguingly, both blank (f) and medicated (g) CPT/CS hybrid NPs thermographies encountered dissimilar exothermic as well as endothermic circumstances, in comparison with individual components. Such differences depend on the chemical nature of the lipid, production circumstances, and interfacial tensions between the organic phase (o) and aqueous phase (w), besides they could be imputed to the development of a new structure with dissimilar thermic features [62]. The shift and overlapping of the melting point of CPT and PF-68 may be due to the small size of hybrid NPs compared to the bulk lipid (CPT), the colloidally dispersed condition of the lipid, and the use of surfactants (PF-68) [68, 69]. Furthermore, the disappearance of APO’s peaks was discerned in the thermography of medicated hybrid NPs suggesting a felicitous encapsulation process inside the hybrid lipid-polymer matrix, concomitant with crystallinity disruption of pure APO. Present data substantiate the FT-IR outcomes and coincide with previously reported ones [19, 21, 22].

### P-XRD studies

The P-XRD scan diffractograms of APO, CPT, PF-68, CS, their PM, lyophilized blank as well as APO-loaded CPT/CS hybrid NPs (F2) are represented in Fig. 4C. APO’s specific crystalline nature (a) was declared by eminent sharp diffraction peaks at 2θ scattered angles of 13.15°, 22.61°, and 26.17° [13]. Both CPT (b) and PF-68 (c) exhibited intense patterns at 21.11°, 23.39° and 19.05°, 23.25°, respectively [51, 57, 70]. Contrariwise, the P-XRD pattern of CS (d) exhibited no sharp distinctive peaks, instead two broad ones at 2θ of 9.93° and 20.09° were observed, typifying its amorphous nature [71]. The P-XRD diffractogram of PM (e) showed an amalgam of the aforenamed intense peaks of CPT and PF-68, whereas those of APO showed a reduced intensity ascribable to the dilution factor. Both blank (f) and medicated (g) CPT/CS hybrid NPs diffractograms exhibited overlapped patterns, with an altered intensity of CPT and PF-68 at 19.03°, 21.23°, and 23.17°, respectively, the dominance of CS amorphous nature, besides the lack of APO’s characteristic diffraction patterns. Thence, encapsulation of APO within the hybrid lipid-polymer matrix in amorphous or molecularly dispersed state exists. Precedently, other drug-loaded hybrid NPs exhibited an analogous approach [19, 52].

### Morphology

**Fig. 5:**
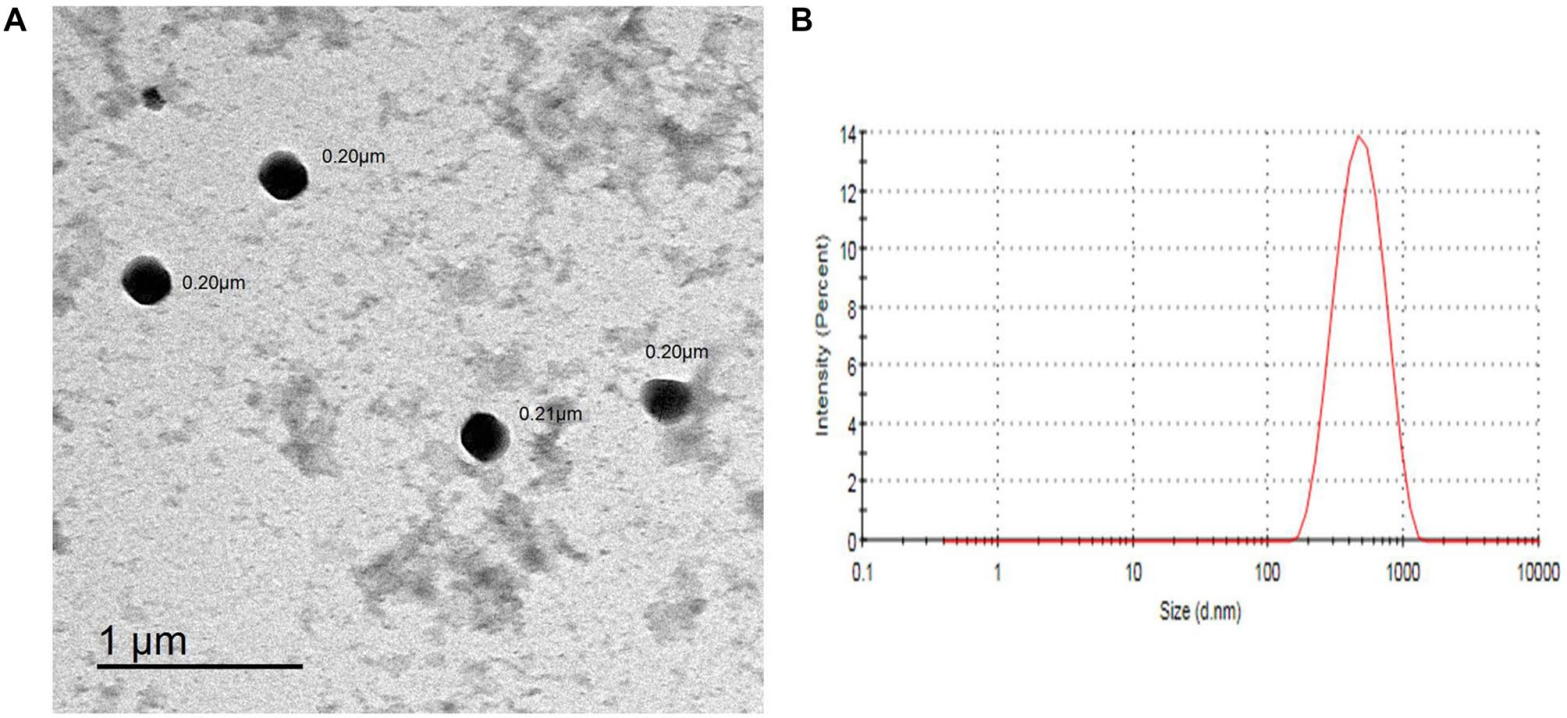
TEM image (**A**) and size distribution curve (**B**) of APO-loaded CPT/CS hybrid NPs (F2)

TEM possesses a significant impact on nanomaterial (NM) structural characterization, using electron diffraction pattern technique, in such a world of science. The TEM micrograph (Fig. 5A) of the optimized formulation (F2) revealed structures with spherical morphology and nanometric size. Moreover, D_h_ value of the photoed formulation, declared by TEM (Fig. 5A), was less than such measured by Zetasizer Nano ZS (Fig. 5B), which could be attributed to the existence of the NPs in the dried form during TEM imaging. Similar observations were precedently declared [22].

### Ex vivo skin permeation study

**Fig. 6:**
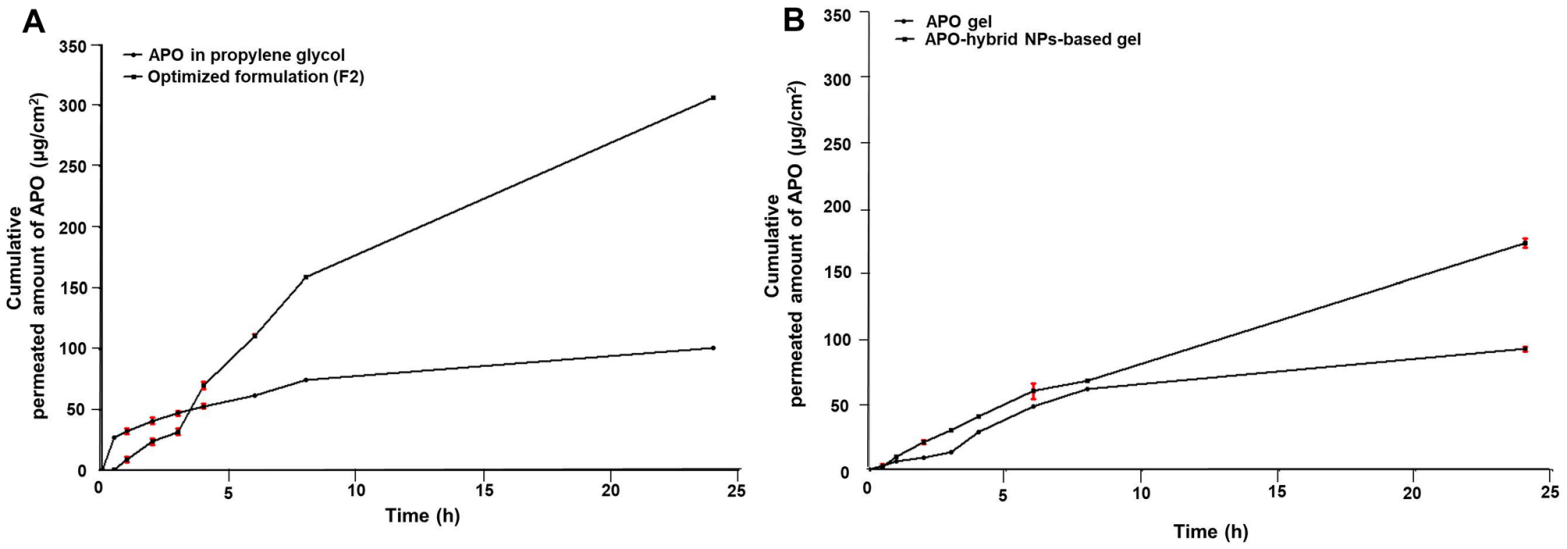
Ex vivo skin permeation profiles of APO from APO-loaded CPT/CS hybrid NPs (F2) in comparison with that of APO in propylene glycol (**A**) and APO-hybrid NPs-based gel in comparison with that of APO gel (**B**)

Aiming to evaluate the optimized formulation (F2) capability of skin targeting besides its permeation ability, an ex vivo skin permeation study through the excised rat skin was performed using locally fabricated horizontal Franz diffusion cells. As illustrated in Fig. 6A, the ex vivo permeation profile of APO from the optimized formulation (F2) was compared with that of the control (drug in propylene glycol). The cumulative permeated amount of APO per unit area from hybrid NPs as well as control formulae were determined during 24-h experiments. Conspicuously, it could be inferred that the optimized formulation (F2) showed a significant difference (*p* < 0.0001) in all the permeation parameters vs the control (Table 3).

**Table 3:** Ex vivo skin permeation parameters of APO from APO in propylene glycol and that from the optimized formulation (F2) across the excised rat skin after 24 h

Formulation	Q_24h_, μg/cm^2^	J_ss_, μg/cm^2^ h	K_p_, cm/h × 10^−3^	ER_flux_
APO in propylene glycol	101.41 ± 1.42	1.99 ± 0.19	2.10 ± 0.20	–––
Optimized formulation (F2)	306.78 ± 2.07^*^	11.10 ± 0.28^*^	11.68 ± 0.29^*^	5.71 ± 0.34

Each value represents the mean ± SD (*n* = 3)

^*^Significant at *p* < 0.0001

### Evaluation parameters of APO gel and APO-hybrid NPs-based gel

The APO gel and APO-hybrid NPs-based gel were assessed for their appearance, color, viscosity, pH, and drug content. The results are presented in Table 4.

**Table 4:** Evaluation parameters of different gel formulations

Parameters	APO gel	APO-hybrid NPs-based gel
Appearance	Transparent homogeneous gel	Opaque homogeneous gel
Color	––––-	White
Viscosity^*^	1127.33 ± 31.75	1262.00 ± 76.96
pH^*^	6.53 ± 0.03	6.69 ± 0.12
Drug content (%)^*^	97.87 ± 1.66	98.29 ± 0.18

^*^Each value represents the mean ± SD (*n* = 3)

### Appearance and color

The APO gel was transparent and homogeneous, while the APO-hybrid NPs-based gel was white, opaque, and homogeneous correlated to the color of the hybrid NPs.

### Viscosity measurement

The viscosity of APO-hybrid NPs-based gel (1262.00 ± 76.96 cP) was slightly higher than that of APO gel (1127.33 ± 31.75 cP), where the hybrid NPs themselves might augment the gel viscosity. Pertinently, the hybrid NPs formulation incorporation into the gel matrix allows easy and consistent delivery of the drug to the targeted site, therefore endorsing better delivery through skin layers [72].

### pH measurement

The pH values of both gels were in the range of 6.53 to 6.69, which is considered reasonable to be applied onto the skin either topically or transdermally [30].

### Drug content

The drug content of APO gel and APO-hybrid NPs-based gel was 97.87 ± 1.66% and 98.29 ± 0.18%, respectively. The results depicted drug content deviation compliance with the official standards of the United States Pharmacopeia. Further, the small values of the SD substantiated a homogenous distribution of APO inside the gel matrix [30].

### Ex vivo skin permeation study for gel formulations

In comparison with APO gel, APO-hybrid NPs-based gel demonstrated a perceptible improved ex vivo permeation profile over a period of 24 h (Fig. 6B) as appraised by significantly (*p* < 0.0001) greater skin permeation parameters with 1.88-, 2.92-, and 2.94-fold increase in Q_24h,_ K_p_, and ER_flux_, respectively, as abridged in Table 5.

**Table 5:** Ex vivo skin permeation parameters of APO from APO gel and that from APO-hybrid NPs-based gel across the excised rat skin after 24 h

Formulation	Q_24h_, μg/cm^2^	J_ss_, μg/cm^2^ h	K_p_, cm/h × 10^−3^	ER_flux_
APO gel	92.30 ± 3.01	2.23 ± 0.13	2.35 ± 0.14	–––
APO-hybrid NPs-based gel	173.54 ± 5.87^*^	6.53 ± 0.09^*^	6.87 ± 0.10^*^	2.94 ± 0.22

Each value represents the mean ± SD (*n* = 3)

^*^Significant at *p* < 0.0001

The enhancement effect of APO-hybrid NPs-based gel in skin permeation might be ascribed to numerous combined reasons enumerated as follows: (1) the nanosized hybrid particles which increase skin-NPs contact and improve drug permeation into the skin; (2) positive ζP value of APO-hybrid NPs loaded in the gel potentially augment permeation besides accumulation in deeper skin areas; (3) the role of nonionic surfactant as a penetration enhancer in solubilizing active ingredients in the stratum corneum (SC) lipid matrix by fluidizing and extracting the lipids in the matrix; (4) the polymeric matrix of APO-hybrid NPs can control drug release, resulting in a high local concentration gradient of the drug and, consequently, its direct transfer from the hybrid NPs to the skin with higher flux values; and (5) hydrophilic character of CP940, with subsequent rapid hydration of skin, enhances the permeation of drug [53, 73–75].

### Kinetic analysis of permeation data

To analyze the mechanism of drug permeation, the coefficients of determination (*R*^2^) and kinetic parameters were reckoned for the optimized formulation (F2) and both gel formulations (Table 6). The ex vivo permeation data for APO gel followed the diffusion-controlled release pattern (Higuchi kinetic model) as the best-fitted model with the highest *R*^2^. Additional scrutiny using Korsmeyer–Peppas mathematical model confirmed a non-Fickian mechanism, 0.5 < *n* < 1, proposing a couple of both erosion and diffusion mechanisms for the drug permeation. Otherwise, the first-order model prevailed for entrapped APO permeation from the optimized formulation (F2) as well as APO-hybrid NPs-based gel matrix, as depicted in Table 6.

**Table 6:** Kinetic analysis of the permeation data of APO from the optimized formulation (F2) and both gel formulations

**Formulation**	**Coefficients of determination (R**^**2**^**)**			**Korsmeyer-Peppas**		
	**Zero order**	**First order**	**Higuchi model**	**(R**^**2**^**)**	**Diffusional exponent (n)**	**Main transport mechanism**
Optimized formulation (F2)	0.9527	0.9586	0.9327	0.9104	------	------
APO gel	0.8528	0.8554	0.9241	0.9349	0.90 ± 0.06	Non-Fickian
APO-hybrid NPs-based gel	0.9823	0.9841	0.9402	0.9374	------	------

### Storage stability study

In terms of stability, the effect of different storage conditions (refrigerated and ambient) on the physical stability of the APO-hybrid NPs-based gel formulation was estimated. No physical changes in appearance, color, and/or presence of any aggregates or lumps were observed in the attested gel formulation after 6 months of storage under different conditions. Similarly, using ANOVA for comparison with the freshly-made preparations of APO-hybrid NPs-based gel, minuscule variation was revealed concerning drug retention % as well as viscosity measurement throughout the specified storage period at the different storage conditions (Table 7). On the other hand, although onto storage under ambient conditions, a significant (*p* < 0.05) decrease in pH value was elucidated which might be due to attendant signs of physical aging of an aqueous dispersion of CP940, the measured value was still physiologically acceptable and suitable for topical as well as transdermal application (Table 7). These findings suggested that loading the hybrid NPs into the gel matrix enhances the physical stability under both refrigerated and ambient conditions. Similar results concerning the storage stability evaluation of lipid-based formulations incorporated into a gel base of CP940 under refrigerated conditions were previously reported [36].

**Table 7:** Drug retention %, viscosity, and pH of APO-hybrid NPs-based gel stored at refrigerated (4 ± 1 °C) and ambient conditions

Storage time	Evaluation parameters					
	Refrigerated conditions (4 ± 1 °C)			Ambient conditions (25 ± 2 °C/60 ± 5% RH)		
	Drug retention (%)	Viscosity	pH	Drug retention (%)	Viscosity	pH
Zero time	100.00 ± 0.00	1262.00 ± 76.96	6.69 ± 0.12	100.00 ± 0.00	1262.00 ± 76.96	6.69 ± 0.12
1 month	98.13 ± 1.68	1247.67 ± 99.85	6.79 ± 0.02	98.47 ± 0.91	1247.67 ± 99.85	6.55 ± 0.01
3 months	98.13 ± 0.40	1248.33 ± 27.54	6.63 ± 0.03	98.13 ± 0.93	1185.33 ± 30.29	6.45 ± 0.02^*^
6 months	97.72 ± 0.43	1207.33 ± 14.19	6.60 ± 0.02	97.69 ± 0.18	1176.00 ± 14.42	6.38 ± 0.03^* a^

Each value represents the mean ± SD (*n* = 3)

^*^Significant at *p* < 0.05 monthly vs. initial

^a^ Significant at *p* < 0.05 refrigerated vs. ambient conditions after 6 months

### Anti-inflammatory efficacy evaluation of APO-hybrid NPs-based gel against CFA-induced RA in rats

Clinically, CFA-induced RA in rats is a validated animal model that is remarkably similar to human RA in both inflammatory and nociceptive complications. As reported previously, CFA causes a series of inflammatory reactions which starts with cutaneous inflammation presented as reddish and swollen at the paw followed by the development of hyperalgesia and edema in the ankle. Thereafter, such acute inflammation extends to affect the adjacent joints as a result of pro-inflammatory cytokines release such as TNF-α besides IL-1β, which also may work together to induce the production of IL-6 in the affected joint synoviocytes. Eventually, the devastating inflammatory reactions result in chronic arthritis [2]. As a consequence, it is considered as a deep-rooted model for inspecting the therapeutic activity of the investigated drugs.

### APO-hybrid NPs-based gel attenuated soft tissue edema and bone deformations

The crucial aim and strategy for RA management are to decrease pain, inflammation and edema, improve joint function and prevent bone deformity and joint destruction [1].

**Fig. 7:**
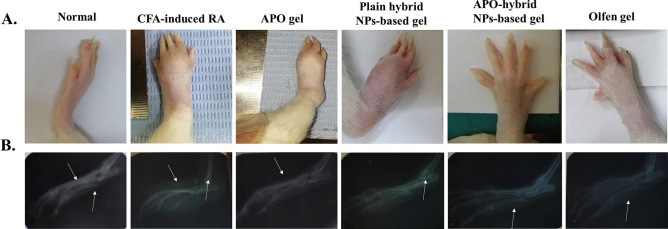
APO-hybrid NPs-based gel formula attenuated soft tissue edema and bone desorption evidenced by **A** paw gross examination and **B** X-ray examination (*n* = 3). Photographic images were taken randomly of each group on the 28^th^ day at the same distance (*n* = 1). The white arrow indicates soft tissue edema

A successful induction of paw edema by CFA sub-plantar injection of the hind paw was confirmed through gross and X-ray examinations (Fig. 7A and B). The CFA-induced RA group exhibited an apparent swelling of the soft tissue of the paw. Moreover, a performed X-ray examination of the ankle joints exhibited soft tissue swelling, narrowing of joint space, and resorption of the bone matrix, in contrast to the normal appearance and bone structure observed in the normal control group. Both plain hybrid NPs-based gel and APO gel groups exhibited a mild ability in subduing these findings. On the other hand, topical treatments in both APO-hybrid NPs-based gel and Olfen^®^ gel groups exhibited the highest decrease in soft tissue swelling and bone resorption.

The renowned merits of both APO and CS are pertinent to the pronounced effect of APO-hybrid NPs-based gel. APO’s anti-inflammatory effect was previously reported in various cell lines and laboratory animal studies of inflammation [6, 76–78]. Preeminently, APO’s anti-inflammatory activity is reinforced upon loading into a novel CPT (lipid)/CS (polymer) hybrid NPs system, where CS is also notorious to exhibit both anti-inflammatory and permeability- ameliorating properties [79, 80]. Furthermore, the enhanced skin permeation of the aforementioned gel formula, which might be ascribed to numerous combined reasons as enumerated in a part of our study (“Ex vivo skin permeation study for gel formulations” section), could strongly help attenuate soft tissue edema and bone desorption.

### APO-hybrid NPs-based gel ameliorated CFA-induced manifestations regarding the changes in paw thickness and body weight

Previous studies have reported defined manifestations associated with CFA-induced edema [81]. Additionally, muscle wasting, also known as cachexia, and body weight loss are known co-morbidity majorly associated with RA progression. Both manifestations are attributed to many factors mostly due to increased oxidative stress, along with cytokine-driven hyper-metabolism and declining in rat feedings [82]. Therefore, the changes in paw thickness as well as body weight, over the course of the experiment, were measured to determine the potential anti-inflammatory and antioxidant activity of APO-hybrid NPs-based gel against CFA-induced paw edema.

**Fig. 8:**
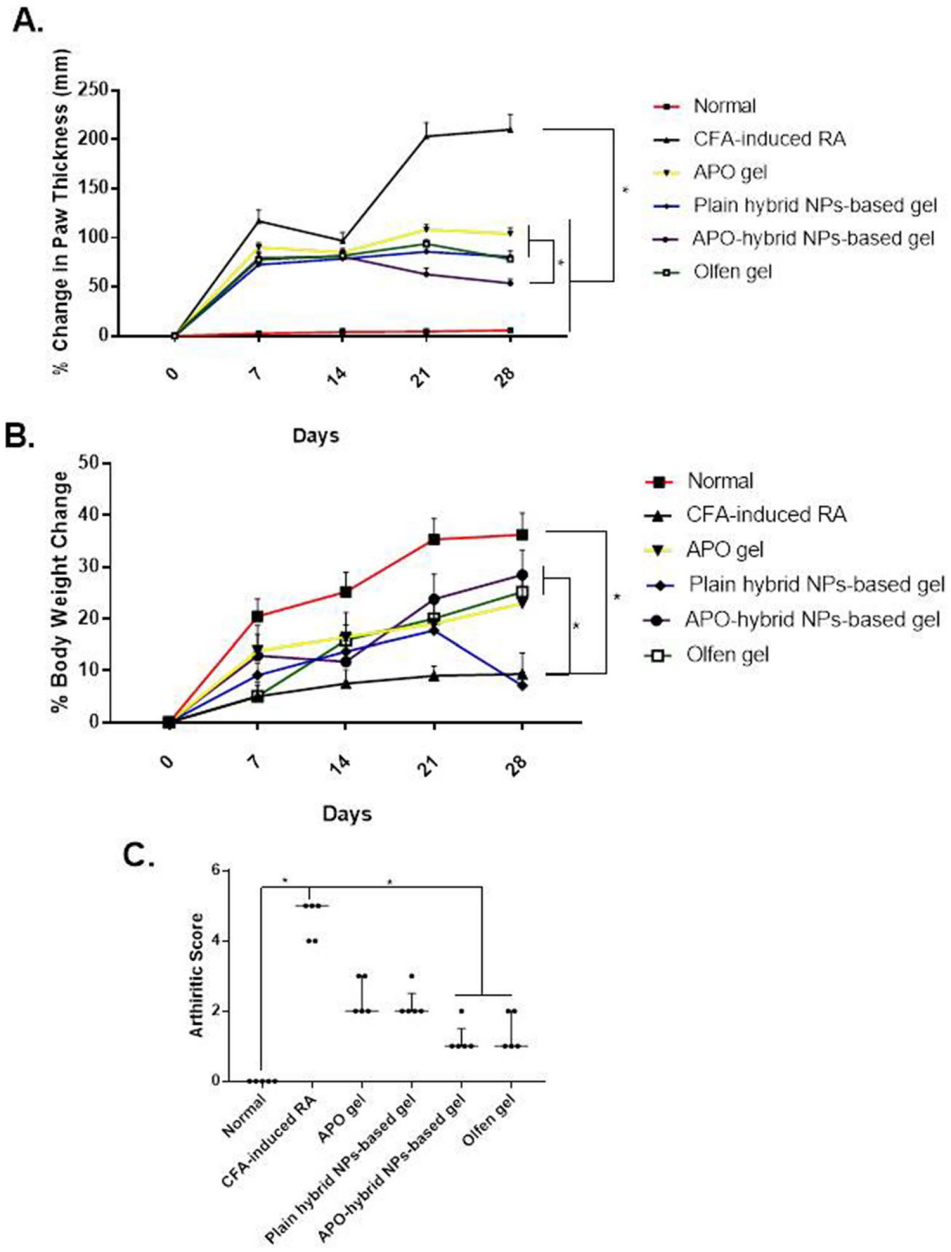
Effect of APO-hybrid NPs-based gel formula on **A** % change in paw thickness, **B** % change in the body weight, and **C** arthritic score, in CFA-induced paw edema. Data values are expressed as mean ± SEM (**A** and** B**) or median and interquartile range (**C**) (*n* = 5). *Significantly different at *p* value < 0.05, using one-way ANOVA followed by Tukey’s multiple comparisons post hoc test (**A** and** B**) and nonparametric using Kruskal–Wallis test followed by uncorrected Dunn multiple comparison test (**C**)

As shown in Fig. 8A, the CFA-induced RA group depicted an increase in the paw thickness over 28 days, as opposed to its analogue the normal control one. Whereas topical treatments of all groups exhibited a significant paw thickness reduction in comparison to the CFA-induced RA group. Discriminatively, reduction in paw thickness was more significantly noticeable in APO-hybrid NPs-based gel-treated group in comparison to the CFA-induced RA one and with either APO- or plain carrier for APO-hybrid NPs-treated groups (Table S2 (Supplementary material) outlines the right paw thickness (mm) of all rats from all the experimental groups assessed on days 0, 7^th^, 14^th^, 21^th^, and 28^th^). Contrariwise, a decline in body weight was evident in the CFA-induced RA group in comparison to the normal control one and was reversed by topical gel treatment in APO gel, APO-hybrid NPs-based gel, and Olfen^®^ gel groups (Fig. 8B).

A significantly high arthritic score, assessed on day 28, was recorded for the CFA-induced RA group, in comparison to the normal control one (Fig. 8C). A significant decrease in that score was observed in both APO-hybrid NPs-based gel and Olfen^®^ gel groups, compared to the CFA-induced RA group. Contrarily, a nonsignificant difference between the CFA-induced RA group and both APO gel- and plain hybrid NPs-based gel-treated groups was detected. Successively, such results established the potentiated anti-inflammatory and antioxidant activity of APO-hybrid NP-based gel formula.

### APO-hybrid NPs-based gel ameliorated CFA-induced manifestations of the hind paw

**Fig. 9:**
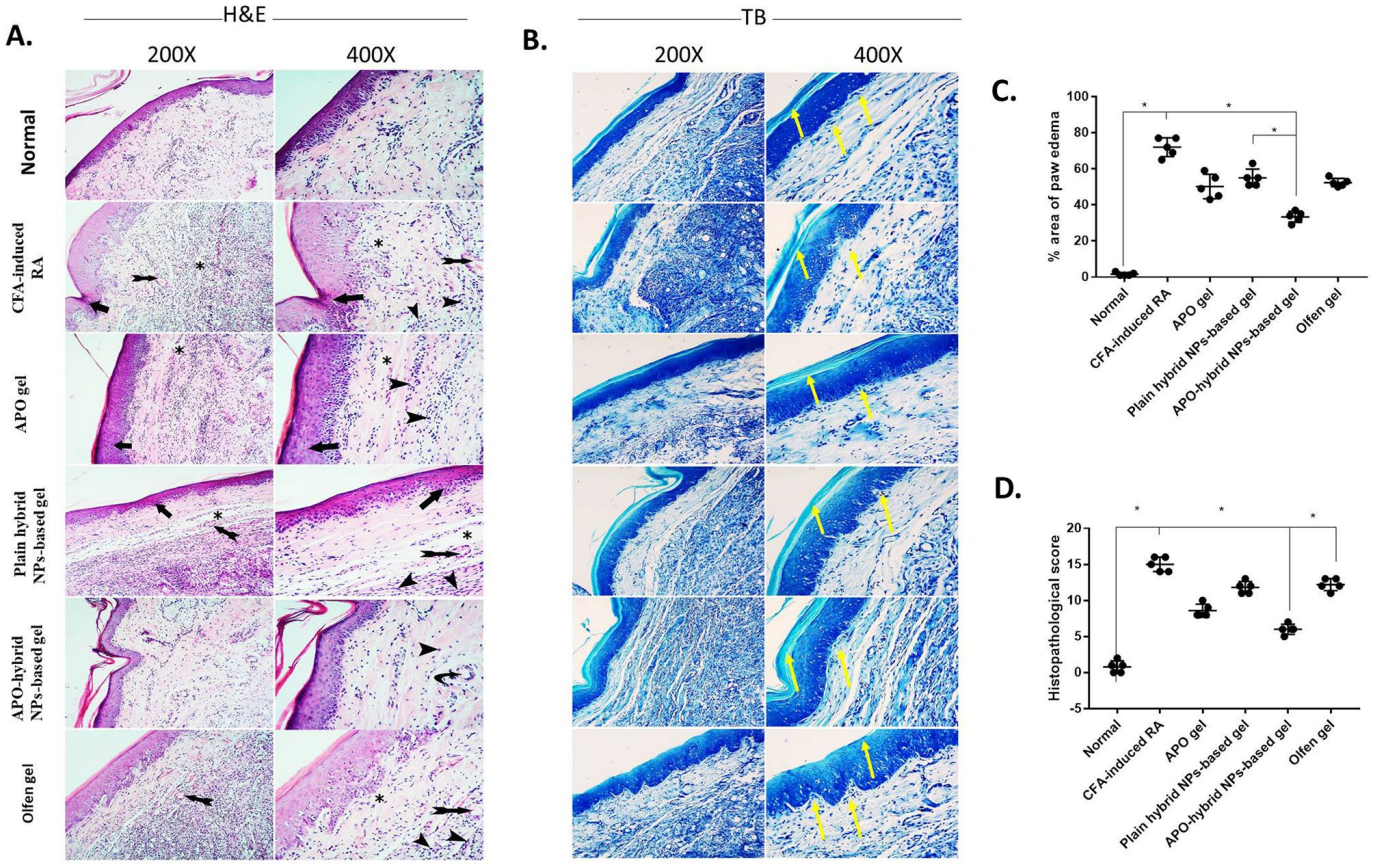
Effect of APO-hybrid NPs-based gel formula on **A** H&E-stained right hind paw tissue, **B** TB-stained right hind paw tissue, **C** % of area paw edema, and **D** histopathological score, in CFA-induced paw edema. Black arrows indicate hyperkeratosis and epithelial proliferation. Asterisk, arrowheads, and tailed arrows indicate marked edema, diffuse inflammatory cells infiltration, and congested vasculature, respectively, in the subcutaneous tissue. The yellow arrow indicates the accumulation and regularity of the proteoglycan deposition in the outer layers. Low magnification × 200 bar; high magnification × 400 bar (**A** and** B**). Data values are expressed as median and interquartile ranges; *significantly different at *p* value < 0.05, using nonparametric Kruskal–Wallis test followed by uncorrected Dunn multiple comparison test (**C** and **D**)

Figure 9 A shows microphotostats, H&E-stained sections, of rats’ hind paws from all experimental groups. The normal control group exhibited a normal histological structure of the epidermis and subcutaneous tissue. On the other hand, the CFA-induced RA group showed severe inflammatory manifestations in the epidermis in the form of hyperkeratosis and epithelial proliferation. In addition, such manifestations were associated with a marked increase in the paw edema (Fig. 9C), histopathological score (Fig. 9D), and diffusion of the inflammatory cells with congested vasculature. Groups treated with either APO gel or plain hybrid NPs-based gel exhibited less marked affection of the epidermal hyperkeratosis as well as epithelial proliferation and minimal edema but with inflammatory cells infiltration and non-congested blood vessels in the subcutaneous tissue. The APO-hybrid NPs-based gel group showed a near-normal epidermis with no edema, scarce inflammatory cells infiltration, and noncongested blood vessels in the subcutaneous tissue. The Olfen^®^ gel-treated group showed a similar effect; however, minimal edema with mild inflammatory cells infiltration and congested blood vessels were present

In Fig. 9B, TB-stained paw tissue sections of the normal control group exhibited normal histoarchitecture. The CFA-induced RA group showed surface irregularities and a decrease in matrix contents with edema and marked inflammatory reaction. Topical application of conventional APO gel, as well as the plain hybrid NPs-based gel, still showed surface irregularities with a less marked decrease of the matrix contents and edema with inflammatory reaction. Both APO-hybrid NPs-based gel- and Olfen^®^ gel-treated groups showed less marked surface irregularities, less marked decrement of the matrix contents, and edema but with inflammatory reaction. Considerably, the histopathological examination was in accordance with the gross and X-ray ones of the different groups, substantiating the potentiated therapeutic anti-inflammatory efficacy of APO-hybrid NPs-based gel formula, that conceivable to be linked to formulation of “anti-inflammatory” APO into a hybrid NPs system based on “anti-inflammatory” CS.

### APO-hybrid NPs-based gel ameliorated CFA-induced extraarticular manifestations of the spleen

**Fig. 10:**
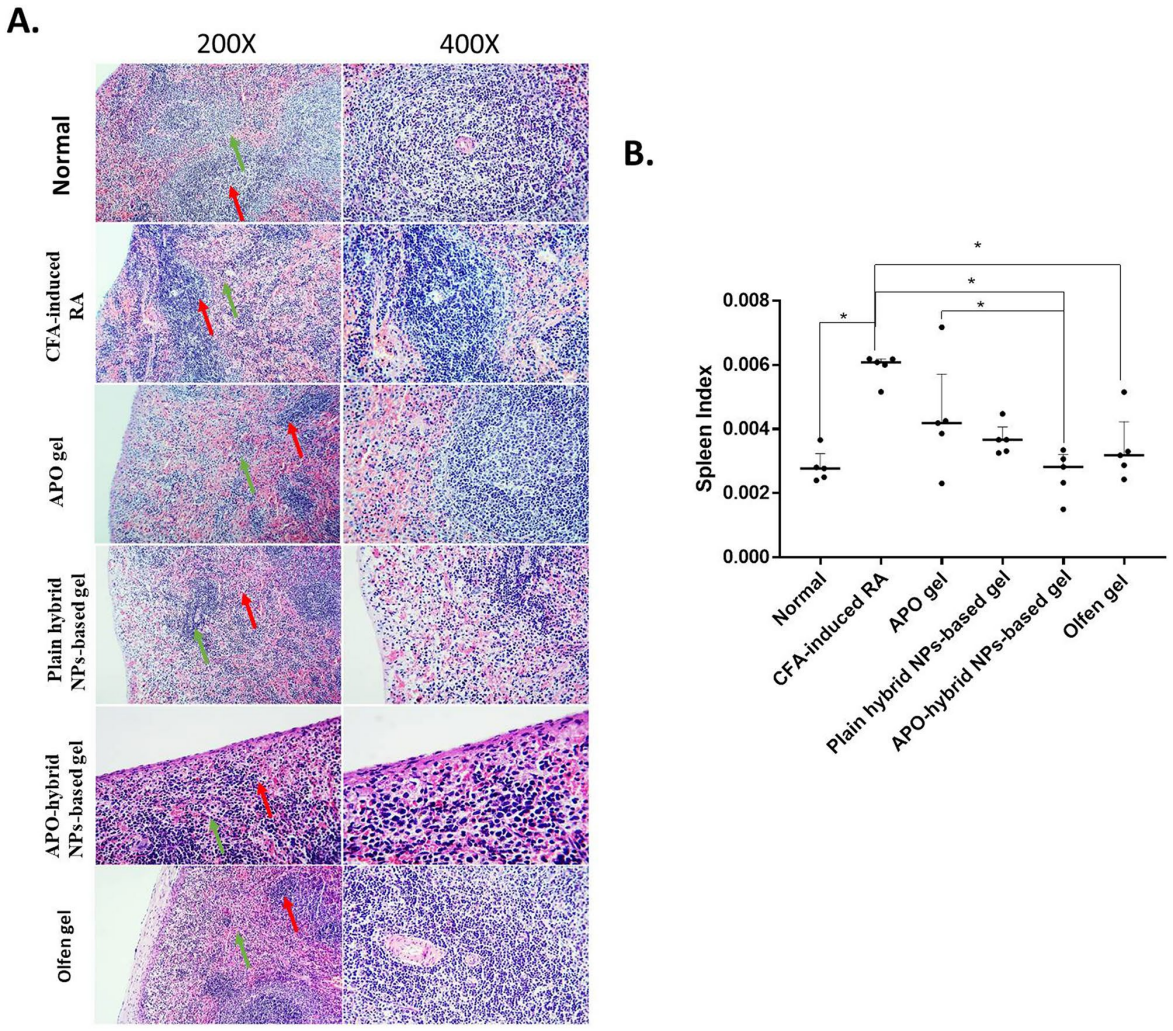
Effect of APO-hybrid NPs-based gel formula on **A** H&E-stained spleen tissue, **B** spleen index in CFA-induced spleen alterations. The green and red arrows indicate white and red pulps, respectively. Low magnification × 200 bar; high magnification × 400 bar (**A**). Data values are expressed as median and interquartile ranges; *significantly different at *p* value < 0.05, using nonparametric Kruskal–Wallis test followed by uncorrected Dunn multiple comparison test (**B**)

The spleen, as a key immune organ, was reported to play an eminent role in the development of the systemic inflammatory process in RA, where progressive changes in the splenic structure associated with CFA-induced RA have been previously noticed. Besides, an increment in the spleen index is commonly observed in such model [83–85]. Therefore, monitoring both the splenic structural changes (Fig. 10A) besides the spleen index changes (Fig. 10B), over the course of the experiment, was used to evaluate the potential anti-inflammatory and antifibrotic effects of APO-hybrid NPs-based gel against CFA-induced RA.

As shown in Fig. 10 A and B, H&E staining of the spleen tissue of the normal control group exhibited a normal histological structure with distinct white and red pulps accompanied by a low spleen index. The CFA-induced RA group showed a visible reduction in the size of the white pulp lymphoid follicles with the expansion of the red pulp, associated with a significant increase in the spleen index. The topical application of APO gel failed to improve such observation. The plain hybrid NPs-based gel and Olfen^®^ gel groups also exhibited marked red pulp expansion with loss of distinction with the white pulp and nearly unidentifiable lymphoid follicles. In contrast, the APO-hybrid NPs-based gel-treated group showed minimal red pulp expansion and preservation of the white pulp.

Topical application of APO-hybrid NPs-based gel successfully ameliorated the splenic manifestations by exerting APO’s reported anti-inflammatory activity [6, 76–78]. Besides, the recently documented antifibrotic effect of APO, a specific NADPH oxidase inhibitor, could limit the production of free radicals, inflammation, and subsequent chronic fibrosis [86].

### APO-hybrid NPs-based gel ameliorated CFA-induced oxidative stress

Oxidative stress, occurring due to an imbalance between prooxidants and antioxidants with consequent excessive production of reactive oxygen species (ROS), is considered a promotor of RA pathogenesis as it contributes to joint tissue damage [87]. CFA is known to provoke such ROS production that further exacerbates arthritis by triggering immune cells to release enzymes and pro-inflammatory cytokines. Besides, the inflammation process also causes oxidative stress as host immune cells, like neutrophils, release large amounts of ROS via the NADPH oxidase enzyme pathway [88]. Oxidative stress-induced via the aforementioned pathway, as a result of CFA injection, has been reported to stimulate both MDA, as a marker of lipid peroxidation, along with NOx, a known product of an augmented pro-inflammatory cytokines release [89, 90]. Previously, the use of phenolic antioxidant phytochemicals to scavenge ROS has been redeemed promising in reducing CFA-induced RA-associated tissue damage [91].

**Fig. 11:**
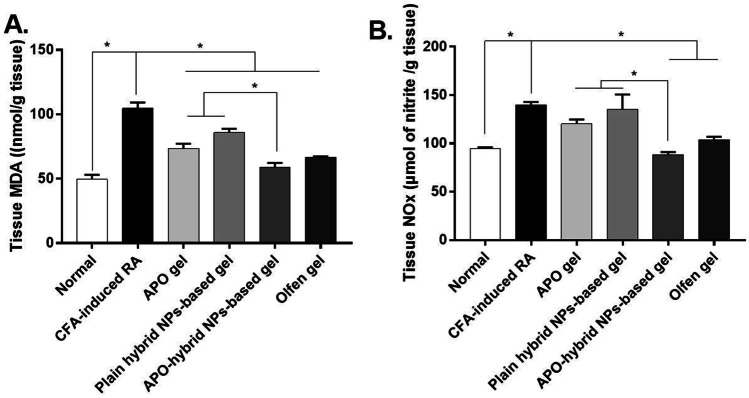
Effect of APO-hybrid NPs-based gel formula on CFA-induced oxidative stress in rats paw tissue. **A** Tissue level of MDA,** B** tissue level of NOx. Data are expressed as mean ± SEM (*n* = 5). *Significantly different at *p* value < 0.05, using one-way ANOVA, followed by Tukey’s multiple comparisons post hoc test

The paw tissue homogenate quantities of oxidative stress biomarkers, namely, MDA and NOx, are shown in Fig. 11 A and B, respectively. Evidently, the CFA-induced RA group showed significant oxidative stress manifested by a significant elevation of both biomarkers levels in comparison to the normal control one. The majority of the treatment groups were able to exhibit a significant reduction in the levels of both markers compared to the CFA group, especially the APO-hybrid NPs-based gel group. Besides, a significant decrease in both MDA and NOx levels was observed in APO-hybrid NPs-based gel group, compared to both APO gel and plain hybrid NPs-based gel ones. Such an enhanced influence could be ascribed to the formulation of a specific plant-based phenolic NADPH oxidase inhibitor “antioxidant APO” into a hybrid NPs system based on “antioxidant and antiarthritic” CS [92, 93].

### APO-hybrid NPs-based gel ameliorated CFA-induced pro-inflammatory release

Serum CRP, an acute-phase inflammatory protein, was firstly measured as an indicator of a general inflammatory incidence. CRP has been reported in CFA-induced RA, releasing the proinflammatory cytokines and stimulating osteoclastogenesis, adding to CFA damaging effect on the paw tissue [94]. Therefore, the paw tissue homogenate levels of pro-inflammatory cytokines, namely, TNF-α, IL-6, and IL-1β, a known trigger of CRP release, were assessed, as well. TNF-α is a central molecule in the augmentation of inflammation via synovial fibroblast stimulation, with subsequent upregulation of other cellular mediators such as the interleukins (ILs), and leukocyte migration enhancement adding to the joint damage effect. IL-6 is known for its damaging effects by stimulating blood vessel growth and promoting inflammation. Additionally, IL-1β has been suggested to regulate bone resorption and cartilage damage as well as, induction of NOx synthesis. Hence, to further verify whether the amelioratory effect of the APO-hybrid NPs-based gel topical treatment of CFA-induced RA is correlated to repression of the inflammation cascade, CRP, TNF-α, IL-6, and IL-1β assessments were carried out.

**Fig. 12:**
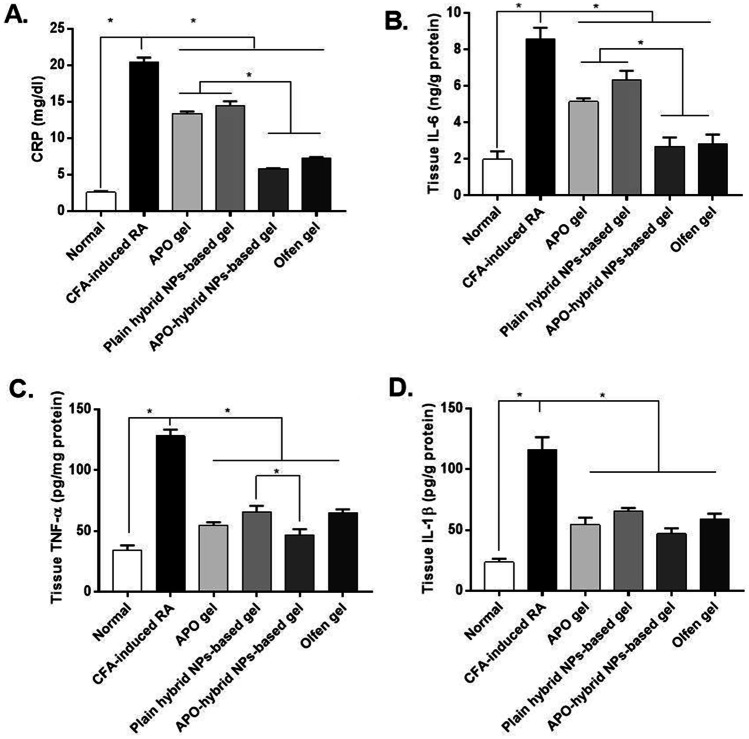
Effect of APO-hybrid NPs-based gel formula on CFA-induced inflammation in rats paw tissue. **A** Serum level of CRP, **B** tissue IL-6, **C** tissue TNF-α, **D** tissue IL-1β. Data are expressed as mean ± SEM (*n* = 5). *Significantly different at *p* value < 0.05, using one-way ANOVA, followed by Tukey’s multiple comparisons post hoc test

In the present study, serum CRP level was elevated in the CFA-induced RA group compared to the normal control one (Fig. 12A). Although, all topical treatments were able to reduce its serum level compared to the CFA-induced RA group, the most significant reduction occurred by APO-hybrid NPs-based gel. Topical application of APO-hybrid NPs-based gel was also able to reduce the paw tissue levels of IL-6 (Fig. 12B), TNF-α (Fig. 12C), and IL-1β (Fig. 12D), compared to the CFA-induced RA and most of the other treated groups. Prominently, APO-hybrid NPs-based gel was as comparable as Olfen^®^ gel in reducing joint tissue levels of IL-6 and IL-1β and TNF-α. Suchlike outcomes declare that the illustrious effect of the APO-hybrid NPs-based gel formulation is pertinent to the well-known anti-inflammatory effects of both APO and CS, with subsequent mitigation of CRP release, besides TNF-α, IL-6, and IL-1β expressions, in case of RA pathogenesis.

Collectively, the preceding in vivo outcomes were agreeable with each other, supporting the anticipated effectual therapeutic activity of the APO-hybrid NPs-based gel formulation against CFA-induced RA. Such prominent therapeutic efficiency could be ascribed to several interrelated factors, which are as follows: (1) the enhanced skin permeation of APO-hybrid NPs-based gel which in turn enhances the permeation of the drug [53, 73–75]; (2) either APO or CS exhibit anti-inflammatory and antioxidant attributes, implying their pharmacological influence on RA-induced manifestations [78–80, 92, 93]; (3) the antifibrotic effect of APO could ameliorate the CFA-induced extraarticular manifestations [86]; (4) the antiarthritic activity of CS can enhance the therapeutic influence of the formulation [93]; (5) positive ζP value of APO-hybrid NPs loaded in the gel, due to the presence of CS, potentially augment gel permeation besides accumulation in deeper skin areas [53]; (6) considering macrophages as phagocytic immune cells which are located in different organs and tissues in chronic inflammatory diseases, NPs can goal them normally and join into their bodies simply with discriminative cumulation in these unhealthy organs [95].

## Conclusion

The contemporaneous study emphasizes the feasible practicability of loading APO, a specific plant-based phenolic NADPH oxidase inhibitor, into a novel CPT (lipid)/CS (polymer) hybrid NPs system via a single emulsion-solvent evaporation technique (o/w). The anti-inflammatory, antioxidant, antiarthritic, and permeability enhancing properties of the used polymer, CS, potentiate the therapeutic activity of APO, upon further formulating into hybrid NPs-based gel formula, against CFA-induced RA in rats. SED approach (fully randomized design (3^2^)) was strictly followed to optimize two IAPs, namely, CPT amount (X_A_) as well as PF-68 concentration (X_B_), for preparing a hybrid NPs system with minimum D_h_ as well as PI, maximum ζP, and EE % value within the range. Furthermore, FT-IR, DSC, and P-XRD outcomes of the optimized formulation (F2) substantiated drug encapsulation in the hybrid matrix. TEM micrograph discloses structures with spherical architecture and nanometric size. Moreover, the developed APO-hybrid NPs-based gel, as a promising topical delivery system, demonstrated a perceptible improved ex vivo permeation profile compared to APO gel. Indubitably, the APO-hybrid NPs-based gel formulation conferred an astounding in vivo therapeutic efficacy against CFA-induced RA in rats compared to APO gel, plain hybrid NPs-based gel, and Olfen^®^ gel. In accordance with the antecedent data, clinical prospective studies are highly required to establish the APO-hybrid NPs-based gel formulation clinical efficacy and give more insight into potential commercial production as an alternative to the currently available RA therapies.

### Supplementary Information

Below is the link to the electronic supplementary material.

Supplementary file1 (PDF 97 KB)

Supplementary file2 (PDF 94 KB)

Supplementary file3 (PDF 130 KB)

Supplementary file4 (PDF 196 KB)

## Data Availability

The datasets generated during and/or analyzed during the current study are available from the corresponding author on reasonable request.
