# Supplementary material for: In vitro–in vivo assessments of apocynin-hybrid nanoparticle-based gel as an effective nanophytomedicine for treatment of rheumatoid arthritis
Source: Drug Deliv Transl Res. 2023 Jun 7;13(11):2903–29. doi: 10.1007/s13346-023-01360-5 (PMC10545657; doi:10.1007/s13346-023-01360-5)
Supplement: Supplementary file 1 — Supplementary file1 (PDF 97 KB) [file 13346_2023_1360_MOESM1_ESM.pdf]

Drug Delivery and Translational Research, Research Article Manuscript

Title:

In vitro-in vivo assessments of apocynin-hybrid nanoparticles-based gel as an effective nanophytomedicine for treatment of rheumatoid arthritis

Authors:

Reham Mokhtar Aman[1]*. Randa Ahmed Zaghloul[2]. Wael M. Elsaed[3]. Irhan Ibrahim Abu Hashim[1]

[1]*Department of Pharmaceutics, Faculty of Pharmacy, Mansoura University, Mansoura, Dakahlia, 35516, Egypt*

[2]*Department of Biochemistry, Faculty of Pharmacy, Mansoura University, Mansoura, Dakahlia, 35516, Egypt*

[3]*Department of Anatomy and Embryology, Faculty of Medicine, Mansoura University, Mansoura, Dakahlia, 35516, Egypt.*

***Corresponding Author:**

Reham Mokhtar Aman, Ph. D.

Lecturer of Pharmaceutics

Department of Pharmaceutics

Faculty of Pharmacy - Mansoura University

El-Gomhoria Street, Mansoura, Dakahlia, 35516, Egypt

Phone : +201005070447 ; Fax : +20502247496

E-mail address : rehamaman@mans.edu.eg

ORCID: https://orcid.org/0000-0002-7525-1766

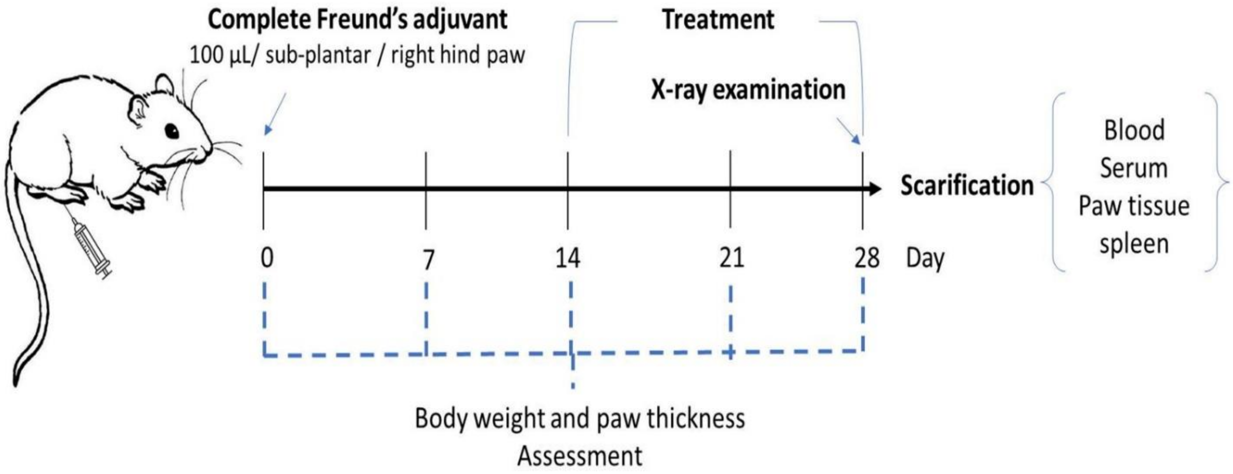

Fig. S1 Schematic representation of the experimental design
