# Supplementary material for: In vitro–in vivo assessments of apocynin-hybrid nanoparticle-based gel as an effective nanophytomedicine for treatment of rheumatoid arthritis
Source: Drug Deliv Transl Res. 2023 Jun 7;13(11):2903–29. doi: 10.1007/s13346-023-01360-5 (PMC10545657; doi:10.1007/s13346-023-01360-5)
Supplement: Supplementary file 2 — Supplementary file2 (PDF 94 KB) [file 13346_2023_1360_MOESM2_ESM.pdf]

Drug Delivery and Translational Research, Research Article Manuscript

Title:

In vitro-in vivo assessments of apocynin-hybrid nanoparticles-based gel as an effective nanophytomedicine for treatment of rheumatoid arthritis

Authors:

Reham Mokhtar Aman[1]*. Randa Ahmed Zaghloul[2]. Wael M. Elsaed[3]. Irhan Ibrahim Abu Hashim[1]

[1]*Department of Pharmaceutics, Faculty of Pharmacy, Mansoura University, Mansoura, Dakahlia, 35516, Egypt*

[2]*Department of Biochemistry, Faculty of Pharmacy, Mansoura University, Mansoura, Dakahlia, 35516, Egypt*

[3]*Department of Anatomy and Embryology, Faculty of Medicine, Mansoura University, Mansoura, Dakahlia, 35516, Egypt.*

***Corresponding Author:**

Reham Mokhtar Aman, Ph. D.

Lecturer of Pharmaceutics

Department of Pharmaceutics

Faculty of Pharmacy - Mansoura University

El-Gomhoria Street, Mansoura, Dakahlia, 35516, Egypt

Phone : +201005070447 ; Fax : +20502247496

E-mail address : rehamaman@mans.edu.eg

ORCID: https://orcid.org/0000-0002-7525-1766

Table S1 Statistical relevance of all IAPs effects either linear, interaction or non-linear on the DRPs

			**D_h (nm)**	**PI**	**ζP (mV)**	**EE %**
IAPs linear effects	CPT amount (X_A)	*P*-value	< 0.0001*	< 0.0001*	< 0.0001*	0.0004*
		Estimate coefficient	**+184.77**	**+0.0865**	**-3.62**	**+1.82**
	PF-68 concentration (X_B)	*P*-value	< 0.0001*	< 0.0001*	0.0190*	< 0.0001*
		Estimate coefficient	**+96.78**	**-0.0482**	**+1.17**	**+2.74**
IAPs interaction effect	CPT amount vs. PF-68 concentration (X_{AB})	*P*-value	< 0.0001*	< 0.0001*	0.0041*	0.1406
		Estimate coefficient	**-64.33**	**-0.0353**	**-1.06**	+0.4525
IAPs non-linear effects	CPT amount (X_A^2)	*P*-value	< 0.0001*	0.9273	0.0020*	0.0137*
		Estimate coefficient	**-135.27**	+0.0015	**+2.85**	**-1.98**
	PF-68 concentration (X_B^2)	*P*-value	< 0.0001*	0.3327	0.0003*	< 0.0001*
		Estimate coefficient	**-102.42**	+0.0162	**+3.60**	**+9.53**
	CPT amount vs. PF-68 concentration ($X_A^2X_B$)	*P*-value	< 0.0001*	< 0.0001*	0.0002*	< 0.0001*
		Estimate coefficient	**-160.67**	**+0.1134**	**-2.64**	**-7.43**
	CPT amount vs. PF-68 concentration ($X_AX_B^2$)	*P*-value	< 0.0001*	< 0.0001*	0.0017*	0.0057*
		Estimate coefficient	**-141.09**	**-0.0922**	**+2.06**	**+1.61**
	CPT amount vs. PF-68 concentration ($X_A^2X_B^2$)	*P*-value	< 0.0001*	0.0009*	0.0197*	0.1436
		Estimate coefficient	**+194.39**	**+0.0807**	**-2.46**	+1.35

*Significant at $p < 0.05$
