# Supplementary material for: In vitro–in vivo assessments of apocynin-hybrid nanoparticle-based gel as an effective nanophytomedicine for treatment of rheumatoid arthritis
Source: Drug Deliv Transl Res. 2023 Jun 7;13(11):2903–29. doi: 10.1007/s13346-023-01360-5 (PMC10545657; doi:10.1007/s13346-023-01360-5)
Supplement: Supplementary file 4 — Supplementary file4 (PDF 196 KB) [file 13346_2023_1360_MOESM4_ESM.pdf]

Drug Delivery and Translational Research, Research Article Manuscript

Title:

In vitro-in vivo assessments of apocynin-hybrid nanoparticles-based gel as an effective nanophytomedicine for treatment of rheumatoid arthritis

Authors:

Reham Mokhtar Aman[1]*. Randa Ahmed Zaghloul[2]. Wael M. Elsaed[3]. Irhan Ibrahim Abu Hashim[1]

[1]*Department of Pharmaceutics, Faculty of Pharmacy, Mansoura University, Mansoura, Dakahlia, 35516, Egypt*

[2]*Department of Biochemistry, Faculty of Pharmacy, Mansoura University, Mansoura, Dakahlia, 35516, Egypt*

[3]*Department of Anatomy and Embryology, Faculty of Medicine, Mansoura University, Mansoura, Dakahlia, 35516, Egypt.*

*__Corresponding Author:__

Reham Mokhtar Aman, Ph. D.

Lecturer of Pharmaceutics

Department of Pharmaceutics

Faculty of Pharmacy - Mansoura University

El-Gomhoria Street, Mansoura, Dakahlia, 35516, Egypt

Phone : +201005070447 ; Fax : +20502247496

E-mail address : rehamaman@mans.edu.eg

ORCID: https://orcid.org/0000-0002-7525-1766

Table S2 Right paw thickness (mm) of all rats from all the experimental groups assessed on days 0, 7th, 14th, 21th, and 28th

Day / Group	**0**	**7**	**14**	**21**	**28**
Normal	2.61	2.63	2.71	2.72	2.78
	2.45	2.51	2.89	2.59	2.77
	2.61	2.71	2.56	2.6	2.69
	2.52	2.69	2.51	2.89	2.61
	2.5	2.48	2.52	2.46	2.55
Plain hybrid NPs-based gel	2.87	5.62	5.21	5.01	4.86
	2.69	4.1	4.65	4.99	5.39
	2.94	4.9	5.1	5.78	5.28
	2.85	4.21	5.1	5.39	4.81
	2.95	5.92	5.51	5.39	5.48
APO-hybrid NPs-based gel	2.65	4.59	4.92	4.51	3.69
	2.69	4.29	4.65	4.64	4.39
	2.65	6.12	5.19	4.62	4.3
	3.01	4.69	5.12	4.39	4.62
	2.36	4.19	4.25	3.56	3.54
Olfen® gel	2.85	5.74	5.39	5.81	5.42
	2.48	4.51	4.67	4.59	4.51
	3.01	5.53	5.21	6.11	5.31
	2.61	4.39	4.78	5.01	4.45
	2.74	4.19	4.81	5.08	4.73
APO gel	2.76	4.99	5.22	5.71	5.31
	2.35	4.53	4.49	4.91	4.39
	2.38	4.46	4.12	4.74	5.09
	2.88	5.29	5.12	6.53	6.21
	2.27	4.69	4.42	4.52	4.81
CFA-induced RA	2.51	5.41	5.26	7.9	7.79
	2.58	6.45	5.11	7.71	7.75

	2.35	5.47	5.12	7.62	8.11
	2.51	4.89	4.75	8.22	8.46
	3.01	5.78	5.11	7.56	7.74
